# Systemic and cardiac pathology induced by a clinically relevant *USP8* activating mutation

**DOI:** 10.1242/dmm.052695

**Published:** 2026-07-22

**Authors:** Tamara González-Costa, Abel Galicia-Martín, Daniel Calle, Lorena Cussó, María Teresa Soto-Navarrete, Xiaoxi Luo, Lucía Méndez-Peralta, Joaquim Grego-Bessa, José Luis de la Pompa

**Affiliations:** ^1^ Intercellular Signalling in Cardiovascular Development and Disease Laboratory, Centro Nacional de Investigaciones Cardiovasculares Carlos III (CNIC), Madrid 28029, Spain; ^2^ CIBER de Enfermedades Cardiovasculares (CIBERCV), Madrid 28029, Spain; ^3^ Advanced Imaging Unit, CNIC, Madrid 28029, Spain

**Keywords:** USP8, Cortisol independent, Musculoskeletal defects, Ocular defects, Cardiac defects

## Abstract

Cushing's disease (CD), the most common endogenous Cushing's syndrome, is caused by activating mutations in the ubiquitin-specific protease 8 (*USP8*) gene. These mutations drive adrenocorticotropic hormone (ACTH)-secreting pituitary adenomas and hypercortisolism. Clinical manifestations include muscle weakness, osteopenia, cataracts and cardiovascular dysfunction. To investigate the pathogenic mechanisms of *USP8* gain of function, we generated a conditional transgenic mouse model expressing human *USP8* (referred to as *hUSP8*) carrying the most prevalent activating mutation in CD adenomas (p.S718del). Systemic expression of *hUSP8^S718del^* in mice induced diffuse corticotroph hyperplasia of ACTH^+^ cells, rather than pituitary microadenomas, and did not lead to hypercortisolemia. Despite preservation of the hypothalamic–pituitary–adrenal axis, transgenic mice developed skeletal muscle atrophy, bone abnormalities, corneal keratitis with cataracts and cardiac dysfunction. Notably, myocardial-specific expression of hUSP8^S718del^ recapitulated cardiac defects seen in mice with ubiquitous expression, demonstrating the direct role of USP8 activation in the heart. These findings show that expression of a clinically relevant *USP8* gain-of-function mutation in mice recapitulates key features of a USP8-associated syndrome. Our results reveal tissue-specific effects of USP8 beyond pituitary tumorigenesis and identify a direct contribution of USP8 activation to cardiac pathology.

## Teaser

**Summary:** *USP8* gain of function drives systemic pathology beyond pituitary effects, directly causing cardiac dysfunction and multi-organ abnormalities independent of hypercortisolism in a clinically relevant mouse model.

## INTRODUCTION

Cushing's disease (CD) is a rare disease with an annual incidence in Europe of 1.2–2.4 cases per million (Etxabe and Vazquez, 1994; van Haalen et al., 2015). CD originates from an adrenocorticotropic hormone (ACTH)-secreting pituitary adenoma (Cushing, 1994), in which ACTH overproduction stimulates excessive cortisol secretion by the adrenal glands, leading to hypercortisolism. CD is the most common form of endogenous Cushing's syndrome (CS) (Castinetti et al., 2012). Patients with CS develop a wide range of symptoms resulting from systemic cortisol exposure, including central obesity, metabolic, cardiovascular and musculoskeletal disease, and increased risk of cataract development, among others (Bertagna et al., 2009; Castinetti et al., 2012; Sharon et al., 2025). They have a 3.3-fold increased risk of mortality (Ragnarsson et al., 2019), primarily due to cardiovascular complications such as hypertension, atherosclerosis, cardiac hypertrophy, cardioembolic stroke and heart failure (Colao et al., 1999; Etxabe and Vazquez, 1994; Faggiano et al., 2003; Mancini et al., 2004; Pivonello et al., 2005; Vassiliadi and Tsagarakis, 2017). Transsphenoidal surgery for pituitary tumor resection is the first-line treatment, achieving remission in 60–90% of cases (Broersen et al., 2018); besides, medical strategies for cortisol correction can alleviate and even reverse clinical comorbidities (Atkinson et al., 2005; Braun et al., 2020). However, increased cardiovascular risk persists in certain patients with CD despite successful long-term remission of hypercortisolism (Colao et al., 1999; Faggiano et al., 2003; Reincke, 2021; van Haalen et al., 2015), with a 2.5-fold increased mortality risk (Ragnarsson et al., 2019).

Recurrent somatic mutations in ubiquitin-specific protease 8 (*USP8*) are found in 36.5% of sporadic CD pituitary adenomas in human patients (Ma et al., 2015; Perez-Rivas et al., 2015; Reincke et al., 2015; Sbiera et al., 2019). These mutations cluster in a hotspot region within exon 14 of *USP8*, which encodes the highly conserved 14-3-3 binding domain (RSYSSP), and primarily affect residues p.S718 and p.P720 (96.1%) (Ma et al., 2015; Sbiera et al., 2019). These mutations prevent binding of the regulatory 14-3-3 protein, thereby enhancing the deubiquitylating activity of USP8 and functioning as activating mutations (Mizuno et al., 2007; Reincke et al., 2015). The 3-bp deletion p.S718del (*USP8^S718del^*) is the most common *USP8* activating mutation, found in ∼25% of patients with CD with such mutations (Sbiera et al., 2019) and exhibits the highest deubiquitylating activity (Reincke et al., 2015). A *de novo* germline mutation in *USP8* was identified in the same hotspot region in a pediatric patient with CD who exhibited not only common CD features but also atypical systemic symptoms beyond pituitary involvement (Cohen et al., 2019). Common symptoms of CD include the presence of ATCH-producing pituitary adenoma and hypercortisolism, overweight, hyperglycemia, hypertension, hirsutism, and lung, kidney and cardiac disease. In addition, the patient showed developmental delay, dysmorphic facial features, skeletal defects and epidermal hyperkeratosis, among other medical conditions (Cohen et al., 2019). Uncommon multiorgan defects suggest a direct role of USP8 in the pathophysiology of the syndrome, beyond pituitary involvement and hypercortisolism. We hypothesize that these defects could arise from altered cellular pathways regulated by the enhanced deubiquitylating activity of USP8 in target tissues. Current mouse models of CD either overexpress corticotropin-releasing hormone (*Crh*) (Dedic et al., 2012; Nishiyama et al., 2022) or rely on subcutaneous and intracranial implantation of ACTH-secreting AtT20 murine pituitary cells (Helseth et al., 1992; Kemeny et al., 2020; Leung et al., 1982; Nishiyama et al., 2022). Unfortunately, these approaches do not genetically mimic human *USP8* (referred to as *hUSP8*) mutations, thereby restricting their utility for mechanistic studies and therapeutic development.

In this study, we generated a conditional transgenic mouse line in which expression of the *hUSP8^S718del^* mutation is controlled by Cre recombinase. This strategy allowed us to assess the tissue-specific effects of *hUSP8^S718del^* activation in epiblast-derived lineages and, independently, in the myocardium. Our model shows that expression in mice of the most common activating *USP8* mutation found in humans induces systemic pathological alterations that parallel key features of the human genetic syndrome (Cohen et al., 2019).

## RESULTS

### Systemic expression of *hUSP8^GOF^* does not induce CD in mice

We generated a conditional transgenic mouse line expressing *hUSP8* carrying the most common activating mutation identified in patients with CD, p.S718del (Reincke et al., 2015; Sbiera et al., 2019). The transgene construct was inserted into the *Rosa26* locus and includes FLAG- and HA-tagged *hUSP8^S718del^*, preceded by a *loxP*-flanked *STOP* cassette and followed by an *IRES-eGFP* reporter (Fig. 1A; Fig. S1A,B). Upon Cre-mediated recombination, the *STOP* cassette was excised, enabling expression of *FLAG-HA-hUSP8^S718del^* and the *GFP* reporter.

**Fig. 1.**
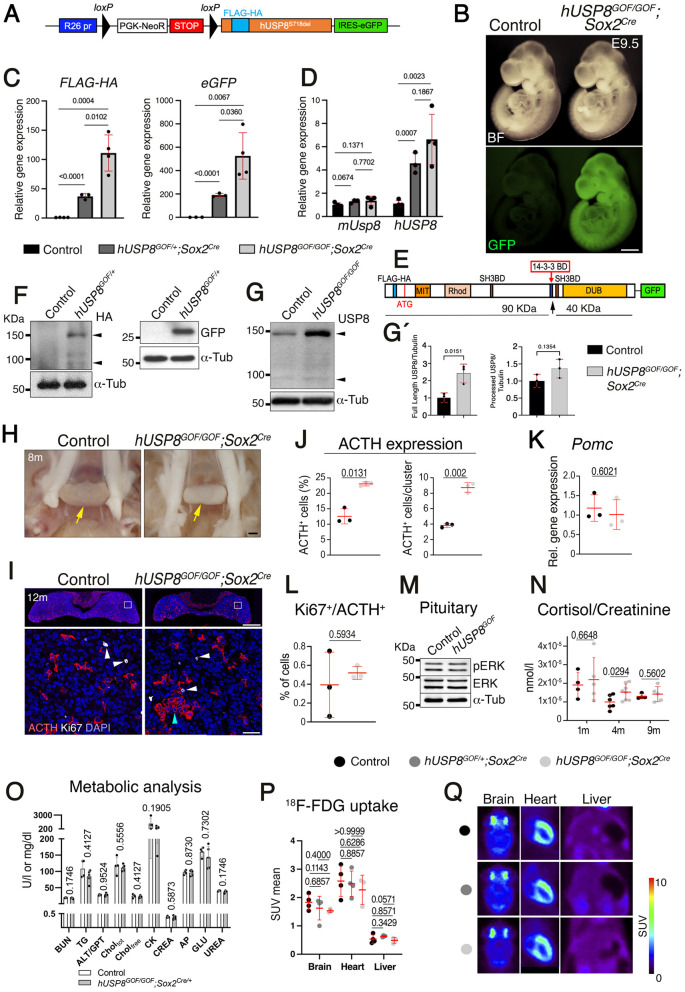
**Design and validation of a CRE-inducible *hUSP8^GOF^* allele.** (A) Schematic representation of the conditional *hUSP8^GOF^* allele regulated by the *Rosa26* promoter, with a floxed *PGK-NeoR-STOP* cassette followed by FLAG-HA-tagged human *USP8^S718del^* (*hUSP8^GOF^*) cDNA and *IRES-eGFP.* Expression is CRE dependent. PGK, NeoR, GFP and IRES-eGFP are indicated. (B) Bright-field and GFP images of embryonic day (E)9.5 control and *hUSP8^GOF/GOF^;Sox2^Cre^* embryos. (C,D) Reverse transcription quantitative real-time PCR (RT-qPCR) for *FLAG-HA*, *eGFP* (C), and mouse (*mUSP8*) and human (*hUSP8*) *USP8* (D) in E11.5 control and transgenic hearts. *FLAG-HA*, *eGFP* and *USP8* were normalized to *Gapdh* expression. Unpaired two-tailed *t*-test. *P*-values are indicated. Data are mean±s.e.m. *n*=3–4 mice/group. (E) Diagram of FLAG-HA-hUSP8^S718del^ protein with domains: FLAG-HA, N-terminal tags in frame with hUSP8; ATG, start codon of the hUSP8 protein; MIT, microtubule interacting and transport domain; Rhod, rhodanese-like domain; SH3BD, SH3 binding domain; 14-3-3 BD, 14-3-3 binding domain (red arrow, hotspot in pituitary adenomas); DUB, deubiquitinase domain; and GFP. Black arrow indicates proteolysis site. (F) Western blot for HA and eGFP in E16.5 control and *hUSP8^GOF/+^;Tnnt2^Cre^* ventricles. α-Tubulin was used as a loading control. Arrowheads indicate specific bands. (G,G′) Western blot (G) and densitometric quantification (G′) for USP8 in E18.5 control and *hUSP8^GOF/GOF^;Sox2^Cre^* hearts. α-Tubulin was used as a loading control. Arrowheads indicate specific bands. Full-length USP8 levels were significantly increased in USP8^GOF^ samples (∼2.3×), whereas the internally processed USP8 fragment showed a non-significant trend toward increase. Protein levels were normalized to α-tubulin. *n*=3–4 mice/group. Data are shown as mean±s.e.m.; *P*-values are indicated above the graphs. Statistical analyses were performed using a two-tailed unpaired *t*-test. (H) Pituitary glands (arrows) from 8-month-old control and *hUSP8^GOF/GOF^;Sox2^Cre^* mice. m, month. (I) ACTH (red), Ki67 (gray) and DAPI (blue) staining in pituitary sections from 1-year-old mice. White arrowheads indicate Ki67^+^ cells. Blue arrowhead indicates ACTH^+^ cluster. Data are mean±s.e.m. *n*=3 mice/group. (J) Quantification of ACTH^+^ cells and ACTH^+^ cells per cluster. Data are mean±s.e.m. *n*=3 mice/group. (K) RT-qPCR for *Pomc* in 1-year-old pituitaries. Data are mean±s.e.m. *n*=3 mice/group. (L) Quantification of ACTH^+^ and Ki67^+^ cells. Data are mean±s.e.m. *n*=3 mice/group. (M) Western blot for total ERK and phosphorylated ERK (pERK) in 1-year-old control and *hUSP8^GOF/+^;Sox2^Cre^* pituitaries. Data are mean±s.e.m. *n*=4–8 mice/group. (N) Urinary cortisol/creatinine ratio at 1, 4 and 9 months in both sexes. Data are mean±s.e.m. *n*=4–8 mice/group. (O) Blood plasma metabolic panel at 9 months: BUN, blood urea nitrogen; TG, triglycerides; ALT/GPT, alanine amino transferase/glutamic-pyruvic transaminase; Chol_tot_, total cholesterol; Chol_free_, free cholesterol; CK, creatine kinase; CREA, creatinine; AP, alkaline phosphatase; GLU, glucose; Urea (calculated as BUN×2.14). Enzyme activity was measured in units (U)/l metabolite concentration in mg/dl. *n*=3–4 mice/group. (P,Q) ^18^F-fluorodeoxyglucose (^18^F-FDG) uptake by positron emission tomography (PET) uptake in brain, heart and liver (P); standardized uptake values (SUV) were color coded as represented (Q). Graphs represent means±s.d. *n*=4 mice/group. Data were analyzed using two-tailed unpaired Student's *t*-test (C,D), two-tailed unpaired Student's *t*-test with Welch's correction (J–N), or two-tailed Mann–Whitney test (O,P), as appropriate. Normality was assessed using the Shapiro–Wilk test. *P*<0.05 was considered statistically significant. Scale bars: 500 μm (B,H,I), 25 μm (insets in I).

To validate conditional expression of the *FLAG-HA-hUSP8^S718del^*::*IRES-eGFP* transgene, we used the *Sox2^Cre^* driver, which induces recombination in epiblast-derived lineages (Hayashi et al., 2002a,b). This approach results in constitutive transgene expression throughout the embryo while excluding extraembryonic tissues (Hayashi et al., 2002b). Whole-mount examination of embryos showed widespread GFP expression in transgenic embryonic day (E)9.5 embryos, indicating that Cre is expressed in all epiblast-derived tissues (Fig. 1B). Given that *FLAG-HA-hUSP8^S718del^* is a gain-of-function (GOF) allele, we refer to mice carrying one copy as *hUSP8^GOF/+^* and those with two copies as *hUSP8^GOF/GOF^*.

To assess transgene expression, we performed reverse transcription quantitative real-time PCR (RT-qPCR) in E11.5 hearts to quantify mRNA levels. Our results confirmed expression of the transgene components, including *FLAG-HA-hUSP8^GOF^* and the reporter *eGFP* (Fig. 1C). Allele-specific expression analysis distinguishing human and mouse *USP8* in transgenic mice showed that *hUSP8* was expressed at levels up to sixfold higher than endogenous murine *Usp8*, consistent with transgene dose-dependent expression (Fig. 1D). The discrepancy between *FLAG-HA*, *GFP* and *USP8* relative gene expression detected by RT-qPCR (Fig. 1C,D) most likely reflects technical differences in assay performance.

The USP8 14-3-3 binding domain is highly conserved between human and mouse (Reincke et al., 2015), supporting functional equivalence across species. The p.S718del mutation disrupts USP8 interaction with 14-3-3 proteins, thereby enhancing proteolytic cleavage of the full-length 130 kDa protein into a 90 kDa N-terminal fragment and a constitutively active 40 kDa C-terminal deubiquitinase (DUB) domain (Reincke et al., 2015). In our *FLAG-HA-hUSP8^GOF^* construct, the HA epitope tag is positioned at the N-terminus of hUSP8^GOF^ (Fig. 1E, light-blue stripe), enabling specific detection by western blot analysis of the full-length ∼130 kDa protein (Fig. 1F, left, upper arrowhead) and an HA-tagged ∼90 kDa N-terminal fragment appearing as a doublet (Fig. 1F, left, lower arrowhead). We could also detect specific transgenic GFP expression (Fig. 1F, right). Western blot analysis of USP8 detected full-length USP8 protein (∼130 kDa; Fig. 1G) that was ∼2.3-fold increased in E18.5 transgenic hearts with respect to that in controls (Fig. 1G′, left). We also observed a lower, 90 kDa band corresponding to the internally processed USP8 fragment (Fig. 1G), the quantification of which showed a non-significant tendency toward increased levels (Fig. 1G′, right). These data show relatively successful expression and post-translational processing of the *FLAG-HA-USP8^S718del^* transgene (Fig. 1E).

One of the hallmarks of CD is the presence of ACTH-secreting pituitary adenomas (Cushing, 1994). To evaluate whether our model recapitulates this aspect, we examined pituitaries from adult transgenic mice. No macroscopic pituitary enlargement was detected in 8-month-old mice expressing two copies of *hUSP8^GOF^* (Fig. 1H). To further characterize the phenotype at the molecular level, we assessed ACTH expression, a key diagnostic marker of CD. Immunofluorescence analysis of pituitaries from 1-year-old *hUSP8^GOF/GOF^;Sox2^Cre^* mice was performed to determine whether USP8 activation leads to increased ACTH production. ACTH-positive cells were detected in both the pars intermedia and pars distalis or adenohypophysis (Fig. 1I), consistent with previous reports (Roux and Dubois, 1976). In control mice, ACTH^+^ cells in the adenohypophysis were typically arranged in small clusters (approximately four cells), whereas *hUSP8^GOF/GOF^;Sox2^Cre^* mice exhibited larger clusters (approximately nine cells) (Fig. 1I,J). Quantitatively, the proportion of ACTH^+^ cells in the adenohypophysis was significantly increased in transgenic mice (23.07±0.66%) compared to that in controls (12.55±2.47%; Fig. 1J). This increase occurred without a corresponding elevation in expression of the ACTH precursor proopiomelanocortin (*Pomc*) (Fig. 1K) or in cell proliferation (Fig. 1I,L), indicating that *hUSP8^GOF^* promotes accumulation of ACTH^+^ cells independently of increased *Pomc* transcription or proliferative expansion. These findings are consistent with enhanced ACTH stability or processing rather than transcriptional upregulation, hyperproliferation or microadenoma formation.

There is increasing evidence that USP8 regulates receptor tyrosine kinase signaling *in vivo* (Niendorf et al., 2007; Pau-Navalon et al., 2026). In *USP8* GOF mutations associated with CD, sustained EGFR signaling leads to increased ERK phosphorylation that drives ACTH hypersecretion and tumor cell proliferation, promoting corticotroph pituirary adenomas (Liu et al., 2019; Reincke et al., 2015). In *hUSP8^GOF/GOF^;Sox2^Cre^* mice, the increase in ACTH^+^ cells did not reflect overactivation of MAPK pathway (Fig. 1M).

Elevated ACTH levels stimulate the hypothalamic–pituitary–adrenal (HPA) axis, leading to increased cortisol production, a hallmark of CD pathophysiology (Bertagna et al., 2009; Castinetti et al., 2012). To assess HPA axis activation in our model, we measured the free cortisol index relative to creatinine in urine samples, a standardized screening for human and canine patients with CD (Nieman et al., 2008; Schafer et al., 2023). Only at 4 months of age, *hUSP8^GOF/GOF^;Sox2^Cre^* mice exhibited a moderate, but significant, increase in cortisol/creatinine levels compared to those of controls (control, 0.99×10^−5^±0.37×10^−5^ nmol/l; transgenic, 1.52×10^−5^±0.43×10^−5^ nmol/l; *P*=0.0294) that reverted to normal levels at 9 months, indicating a mild and transient alteration of the HPA axis (Fig. 1N). Patients with CD frequently develop cardiometabolic syndrome as a consequence of sustained hypercortisolism (Pivonello et al., 2005). To determine whether this phenotype is recapitulated in our model, we analyzed plasma metabolites and enzyme levels in 4- and 9-month-old *hUSP8^GOF/GOF^;Sox2^Cre^* mice and found no significant differences compared to those in age-matched controls (Fig. 1O; Fig. S2D). USP8 has been implicated in tumor progression and cell migration across multiple cell types (Jiang et al., 2025; Ke et al., 2025; Wu et al., 2025; Zhao et al., 2022). To assess potential metabolic alterations associated with increased glucose demand, we measured ^18^F-fluorodeoxyglucose (^18^F-FDG) uptake by positron emission tomography (PET) in the brain, heart and liver. FDG, a glucose analog, accumulates in tissues with elevated metabolic activity, such as tumors, inflammatory lesions, or ischemic myocardium (Pantel et al., 2024). Transgenic mice exhibited FDG uptake levels comparable to those of controls in all organs analyzed (Fig. 1P,Q). Together, these results indicate that *hUSP8^GOF^* expression does not induce overt metabolic or biochemical abnormalities associated with tumorigenesis, inflammation or ischemia in major organs. Chronic lung and kidney disease, as well as adrenal gland hyperplasia due to prolonged dysregulation of the HPA axis, are frequently observed in human patients with CD (Cohen et al., 2019; Reincke et al., 2015). To determine whether these pathologies are recapitulated in our model, we performed histopathological analyses of the lungs, kidneys and adrenal glands in *hUSP8^GOF/GOF^;Sox2^Cre^* mice. Tissue architecture in all examined organs was indistinguishable from that of control mice (Fig. S2A–C). Collectively, these findings indicate that germline expression of *hUSP8^S718del^* in mice is insufficient to induce the classical pathological features of CD.

### *hUSP8^GOF^* induces musculoskeletal defects

*hUSP8^GOF/GOF^;Sox2^Cre^* mice were viable and survived to adulthood but appeared visibly smaller than their control littermates (Fig. 2A). By 1 month of age, homozygous mice exhibited a marked reduction in body weight that persisted for at least 1 year. In contrast, heterozygous *hUSP8^GOF/+^;Sox2^Cre^* mice showed only a mild decrease in body weight (control, 16.7±2.2 g; *hUSP8^GOF/+^;Sox2^Cre^*, 17.4±1.8 g; *hUSP8^GOF/GOF^;Sox2^Cre^*, 12.2±1.0 g; Fig. 2B), suggesting a gene dosage effect of the *hUSP8^GOF^* allele. Proximal myopathy and muscle atrophy are common and clinically relevant comorbidities common in patients with CD and the *USP8* genetic syndrome (Cohen et al., 2019; Mancini et al., 2004; Reincke, 2021; Reincke et al., 2015). To assess skeletal muscle structure, we examined the lateral gastrocnemius (LG) in 1-year-old mice (Fig. 2C). All genotypes showed normal skeletal muscle tissue architecture, with comparable fiber shape and peripheral nuclear localization (Fig. 2; Fig. S3A). However, cross-sectional area analysis revealed a dose-dependent reduction in muscle fiber caliber in *hUSP8^GOF^* mice (control, 8140±454.7 μm^2^; *hUSP8^GOF/+^;Sox2^Cre^*, 4641±2154 μm^2^; *hUSP8^GOF/GOF^;Sox2^Cre^*, 3834±773.4 μm^2^; Fig. 2E), consistent with muscle atrophy in the absence of fibrotic scarring (Fig. S3B). Because the MAPK pathway regulates muscle growth and remodeling (Brennan et al., 2021), we assessed its activity in skeletal muscle. We observed increased phosphorylated ERK (pERK) in *hUSP8^GOF^* muscle (Fig. 2F), indicating elevated pathway activity. These results suggest that hUSP8 overactivation is associated with MAPK signaling changes that may contribute to muscle atrophy. This is consistent with prior studies linking sustained ERK activation to impaired myofiber maintenance and reduced fiber size (Penna et al., 2010), supporting a role for USP8 signaling in muscle homeostasis.

**Fig. 2.**
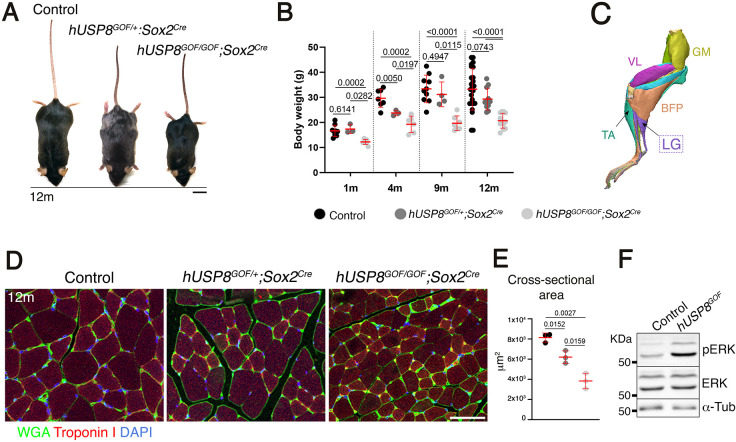
***hUSP8^GOF^* expression induces muscle atrophy.** (A) External view of control and transgenic mice. (B) Body weight tracking from 1 to 12 months in transgenic mice. Data were analyzed using two-tailed unpaired Student's *t*-test with Welch's correction following the Shapiro–Wilk normality test. *n*=3–24 mice per genotype, as indicated in the graph. (C) 3D schematic of hindlimb muscles: LG, lateral gastrocnemius; TA, tibialis anterior; GM, gluteus maximus; VL, vastus medialis; BFP, posterior biceps femoralis [adapted from Charles et al. (2016) under the terms of the CC-BY 4.0 license]. (D) Immunofluorescence of LG muscle cross-sections from 1-year-old control, *hUSP8^GOF/+^;Sox2^Cre^* and *hUSP8^GOF/GOF^;Sox2^Cre^* mice. Wheat germ agglutinin (WGA; green), troponin I (red), DAPI (blue). (E) Quantification of muscle cross-sectional area. Graphs show mean±s.d. Data were analyzed using two-tailed unpaired Student's *t*-test with Welch's correction following the Shapiro–Wilk normality test. *n*=3 biologically independent mice per genotype. Scale bars: 1 cm (A); 50 μm (D).

Patients with CD develop osteopenia and osteoporosis (Castinetti et al., 2012; Di Somma et al., 2002; Naguib et al., 2023), whereas *USP8* syndrome has been associated with dysmorphic facial features and scoliosis (Cohen et al., 2019). One year-old *hUSP8^GOF/GOF^;Sox2^Cre^* mice displayed a visibly misshaped snout compared to that of controls (Fig. S3C). Morphometric analysis using X-ray computed tomography (CT; Fig. 3A,B; Movie 1) revealed a reduction in skull length (SL) and skull width (SW) in these mice (SL: control, 22.24±1.03 mm; *hUSP8^GOF/+^;Sox2^Cre^*, 21.20±0.93 mm; *hUSP8^GOF/GOF^;Sox2^Cre^*, 20.28±0.63 mm; SW: control, 12.72±0.28 mm; *hUSP8^GOF/+^;Sox2^Cre^*, 12.51±0.29 mm; *hUSP8GOF^/GOF^;Sox2^Cre^*, 12.14±0.18 mm), whereas the inner intercanthal distance (IID) remained unchanged (control, 5.10± 0.17 mm; *hUSP8^GOF/+^;Sox2^Cre^*, 5.03±0.17 mm; *hUSP8^GOF/GOF^;Sox2^Cre^*, 5.13±0.16 mm; Fig. 3C). Transgenic mice also showed a tendency towards dorsal kyphosis (control, 157.7±6.09 mm; *hUSP8^GOF/+^;Sox2^Cre^*, 156.4±7.44 mm; *hUSP8^GOF/GOF^;Sox2^Cre^*, 149.7±11.52 mm) and, to a lesser extent, cervical kyphosis (control, 141.50±10.41 mm; *hUSP8^GOF/+^;Sox2^Cre^*, 130.20±12.95 mm; *hUSP8^GOF/GOF^;Sox2^Cre^*, 133.00±27.65 mm; Fig. 3D,E). In addition, *hUSP8^GOF/GOF^;Sox2^Cre^* mice showed a trend toward reduced bone density (control, 3141±422.1 Hounsfield units (HU); *hUSP8^GOF/+^;Sox2^Cre^*, 2980±433.1 HU; *hUSP8^GOF/GOF^;Sox2^Cre^*, 2870±315.4 HU; Fig. 3F). Collectively, *USP8^GOF^* transgenic mice phenocopied human germinal expression of the *USP8* activating mutation leading to musculoskeletal abnormalities (Cohen et al., 2019).

**Fig. 3.**
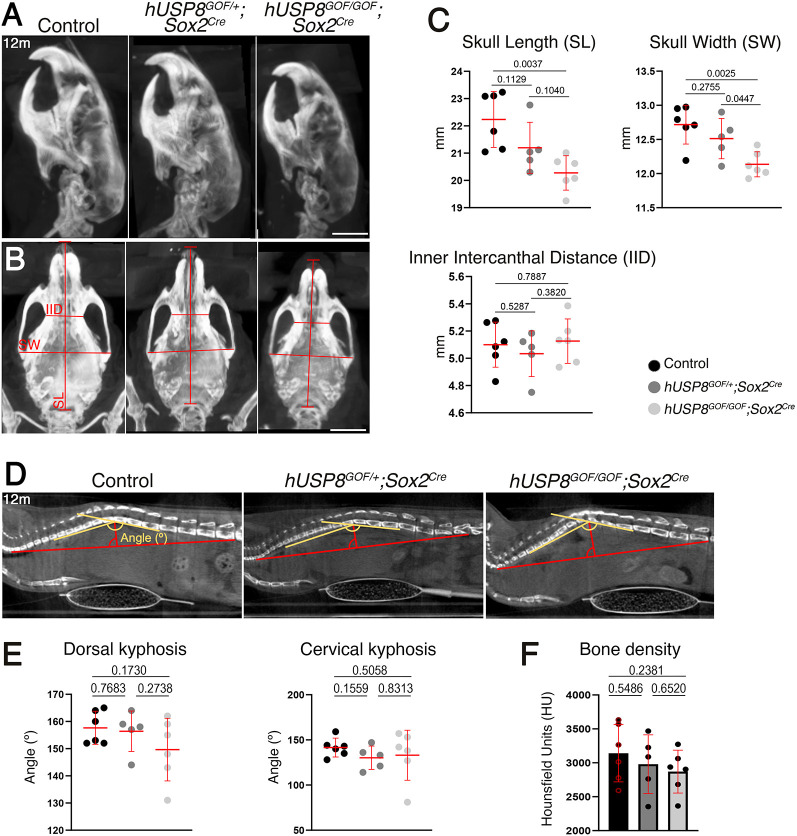
***hUSP8^GOF^* expression induces skeletal defects.** (A,B) X-ray computed tomography (CT)-scan analysis of transgenic skulls showing cranial morphology in lateral and coronal views. SL, skull length; SW, skull width; IID, inner intercanthal distance. (C) Morphometric analysis of SL, SW and IID. (D) Sagittal CT reconstruction of the vertebral column highlighting dorsal kyphotic curvature and quantified angulation (yellow). (E) Quantification of spinal and cervical kyphosis angles. (F) Mean femur bone density measured in Hounsfield units (HU). Graphs show mean±s.d. Data were analyzed using two-tailed unpaired Student's *t*-test with Welch's correction following the Shapiro–Wilk normality test. *n*=5–6 biologically independent mice per genotype. Scale bars: 5 mm.

### *hUSP8^GOF^* induces dose-dependent ocular degeneration

An unexpected phenotype in transgenic mice was the development of ocular opacification, which became apparent at ∼3 months of age and progressively worsened over time. By 1 year of age, ∼50% of *hUSP8^GOF/GOF^;Sox2^Cre^* mice and 20% of *hUSP8^GOF/+^;Sox2^Cre^* mice exhibited visible corneal opacity (Fig. S4A–C). Histological analysis of sagittal eye sections from 1-year-old mice revealed pronounced corneal abnormalities, including thickening and keratinization of the outer stratified squamous epithelium, stromal fiber disorganization, fibrosis, cellular infiltration and neovascularization. (Fig. 4A; Fig. S4D). Detailed histopathological scoring confirmed corneal defects in all *hUSP8^GOF/GOF^;Sox2^Cre^* mice (three of three) and in most heterozygous mice (two of three) at this age (Fig. 4B), suggesting a *hUSP8^GOF^* dose-dependent effect.

**Fig. 4.**
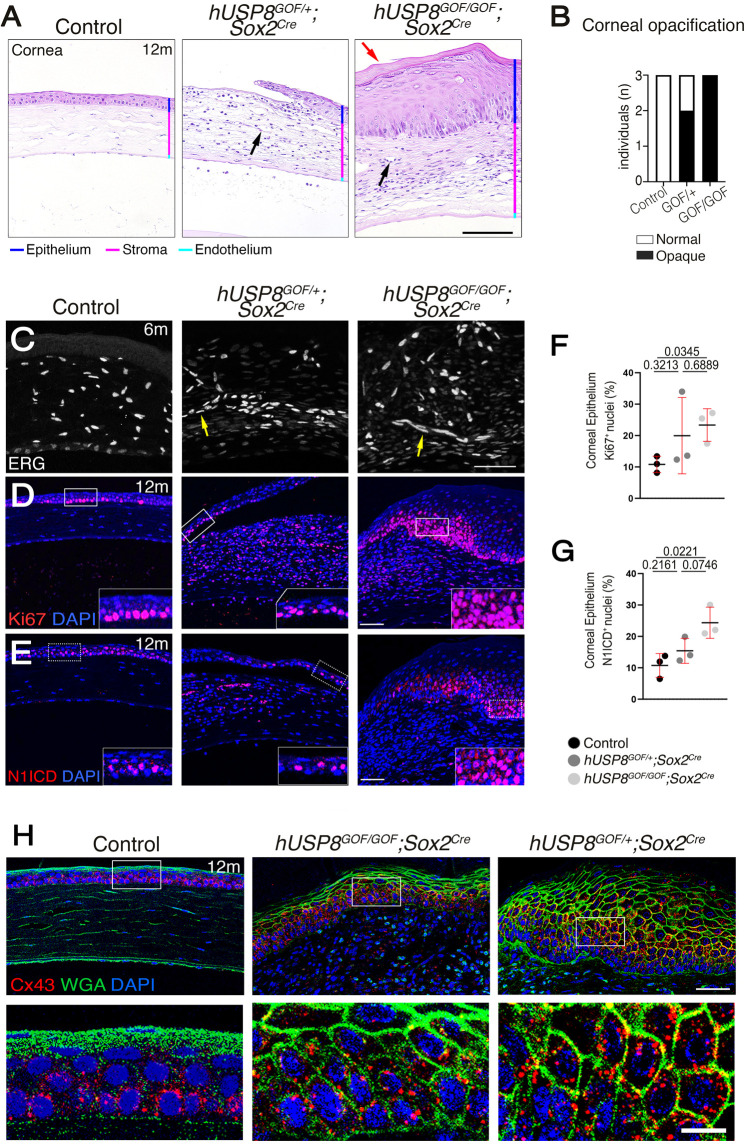
**Corneal defects in *hUSP8^GOF^* mice.** (A) Hematoxylin and Eosin (H&E) staining of sagittal corneal sections from 1-year-old control, *hUSP8^GOF/+^;Sox2^Cre^* and *hUSP8^GOF/GOF^;Sox2^Cre^* mice, showing stromal disarray, cell infiltration, neovascularization (black arrows) and epithelial keratinization (red arrow). (B) Quantification of corneal opacification severity (*n* individuals). (C) ERG immunofluorescence in 6-month-old eye sections; arrows indicate elongated cell clusters. (D) Ki67 (red) and (E) N1ICD (red) immunofluorescence in corneal epithelium at 1 year. (F,G) Quantification of Ki67^+^ (F) and N1ICD^+^ (G) nuclei. (H) Immunofluorescence of CX43 (red), WGA (green) and DAPI (blue) in corneal sections. Graphs show mean±s.d. Data were analyzed using two-tailed unpaired Student's *t*-test with Welch's correction following the Shapiro–Wilk normality test. *n*=3 mice per genotype. Scale bars: 100 μm (A); 50 μm (C–E,H); 10 μm (H, insets).

Corneal transparency relies on the maintenance of an avascular stroma and organized collagen fiber architecture (Teixeira, 2019). In 6-month-old *hUSP8^GOF^* transgenic mice, we observed stromal endothelial cells arranged in elongated clusters, an organization suggestive of active angiogenesis (Fig. 4C). Because epithelial hyperplasia can also contribute to corneal opacification and plaque formation (Vauclair et al., 2007), we assessed cellular proliferation in the corneal epithelium. Ki67 (also known as MKI67) immunostaining revealed significantly increased proliferation in *hUSP8^GOF/GOF^;Sox2^Cre^* corneas (23.36±5.20%) compared to that in controls (10.82±2.61%), particularly in the basal and suprabasal epithelial layers (Fig. 4D,F).

Previous studies have shown that, in cancer cells, USP8 can stabilize and deubiquitylate the active form of the NOTCH1 receptor [NOTCH1 intracellular domain (N1ICD)] via direct interaction (Shin et al., 2020). The NOTCH signaling pathway is also known to regulate cell fate and proliferation, playing a key role in corneal homeostasis and wound healing (Vauclair et al., 2007). Consistent with this, we detected increased N1ICD expression in the basal epithelial layer of transgenic corneas (Fig. 4E), with the strongest activation in homozygous hUSP8^GOF^ mice (24.35±4.97%), followed by heterozygotes (15.40±3.98%) and controls (10.74±3.80%; Fig. 4G). In addition, USP8 is known to regulate the intracellular trafficking of the gap junction protein connexin 43 (CX43) in various cell types (Sun et al., 2018). CX43 is normally expressed in the corneal epithelium (Zhai et al., 2014) and is upregulated and mislocalized during corneal wound healing (Grupcheva et al., 2012). We observed CX43 upregulation and mislocalization in diseased corneas of *hUSP8^GOF/GOF^;Sox2^Cre^* mice, along with marked epithelial hypertrophy (Fig. 4H; Fig. S4D,E). Together, these findings indicate that *hUSP8^GOF^* expression activates a molecular and cellular program in the cornea that closely resembles a chronic wound-healing response.

A healthy ocular lens is avascular, and its inner cortex consists of densely packed and ordered fiber cells, essential for lens transparency (Teixeira, 2019). Histological analysis of lenses from 1-year-old mice revealed early subcapsular deposits, with nuclear retention in *hUSP8^GOF/+^;Sox2^Cre^* mice. These abnormalities were more severe in *hUSP8^GOF/GOF^;Sox2^Cre^* mice, extending into the inner cortex and indicating progressive cataract formation (Fig. 5A,B; Fig. S4D). Cataracts were observed in 100% of the transgenic mice analyzed (six of six), highlighting a fully penetrant lens phenotype (Fig. 5B). These findings uncover a previously unrecognized role of *hUSP8^GOF^* in ocular integrity and aging-related degeneration. Interestingly, recent studies have shown that patients with CD have a 39% higher risk of developing cataracts than the general population (Sharon et al., 2025).

**Fig. 5.**
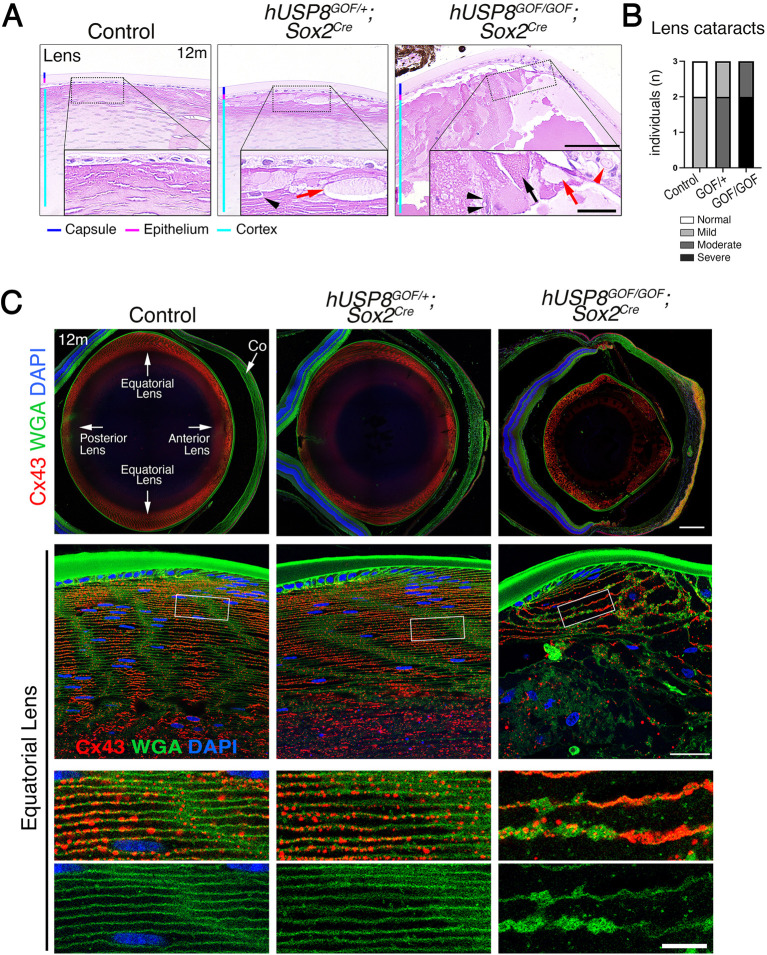
**Expression of *hUSP8^GOF^* induces lens cataracts formation.** (A) H&E staining of lens sections, showing deposits (red arrows), nuclear retention (black arrowheads), fiber disorganization (black arrow) and epithelial inflammation (red arrowhead) in *USP8^GOF/+^;Sox2^Cre^* and *hUSP8^GOF/GOF^;Sox2^Cre^* corneas. Color-coded bars indicate corneal layers. (B) Quantification of lens cataracts severity (*n* individuals). (C) CX43 (red), WGA (green) and DAPI (blue) staining in sagittal eye sections; Co, cornea. Graphs show mean±s.d.; unpaired two-tailed Student's *t*-test. Scale bars: 100 μm (A, insets); 500 μm (C, low magnification); 50 μm (C, high magnification and insets).

CX43 plays a crucial role in maintaining lens avascularity by enabling intercellular communication and metabolic exchange among fiber cells (Goodenough et al., 1980). CX43 is known to be upregulated in ∼50% of age-related cataracts in humans (Gong et al., 2021). To assess its expression and localization, we performed immunofluorescence analysis using CX43 and the membrane marker wheat germ agglutinin (WGA) in adult lenses. In control and heterozygous mice, CX43 was localized to the membranes of well-organized fiber cells; in contrast, *hUSP8^GOF/GOF^;Sox2^Cre^* lenses exhibited disrupted fiber cell architecture and marked CX43 mislocalization (Fig. 5C; Fig. S4F). These data indicate that cataract severity is dose dependent on *hUSP8^GOF^* expression and that altered lens cytoarchitecture and mislocalization of gap junctions contribute to cataractogenesis. Notably, the retina appeared histologically unaffected in transgenic mice (Fig. S4G,H).

### *hUSP8*^*GOF*^ induces structural abnormalities and impaired heart function

*USP8* syndrome is associated with persisting cardiac dysfunction even after transsphenoidal surgery and normalization of cortisol levels (Cohen et al., 2019), consistent with the increased cardiovascular risk observed in patients with CD after successful treatment (Colao et al., 1999; Faggiano et al., 2003). These observations suggest a potential cortisol-independent role for USP8 in cardiovascular homeostasis. To investigate the specific effects of *hUSP8^GOF^* in the myocardium, we generated mice expressing *FLAG-HA-hUSP8^S718del^*::*IRES-eGFP* specifically in cardiomyocytes using the *Tnnt2^Cre^* driver allele, active from E8.0 onwards (Jiao et al., 2003). We examined *GFP* expression at E9.5 (Fig. 6A) and confirmed the restricted myocardial expression of the transgene. Transgenic *hUSP8^GOF/GOF^;Tnnt2^Cre^* mice were viable and survived to adulthood. Longitudinal heart sections from 1-year-old control, *hUSP8^GOF/GOF^;Sox2^Cre^* and *hUSP8^GOF/GOF^;Tnnt2^Cre^* mice revealed normal cardiac anatomy and no signs of fibrosis (Fig. S5A–C). Heart weight was similar across genotypes (Fig. S5D); however, *hUSP8^GOF/GOF^;Sox2^Cre^* mice exhibited an increased heart weight-to-body weight ratio, attributable to their reduced overall body size (Fig. S5E). RT-qPCR confirmed expression of the transgene components *FLAG-HA-hUSP8^GOF^* and the reporter *eGFP* (Fig. S5F). Allele-specific analysis showed that *hUSP8* expression in *hUSP8^GOF/GOF^;Tnnt2^Cre^* transgenic mice reached up to fourfold higher levels than those for endogenous murine *Usp8* (Fig. S5G).

**Fig. 6.**
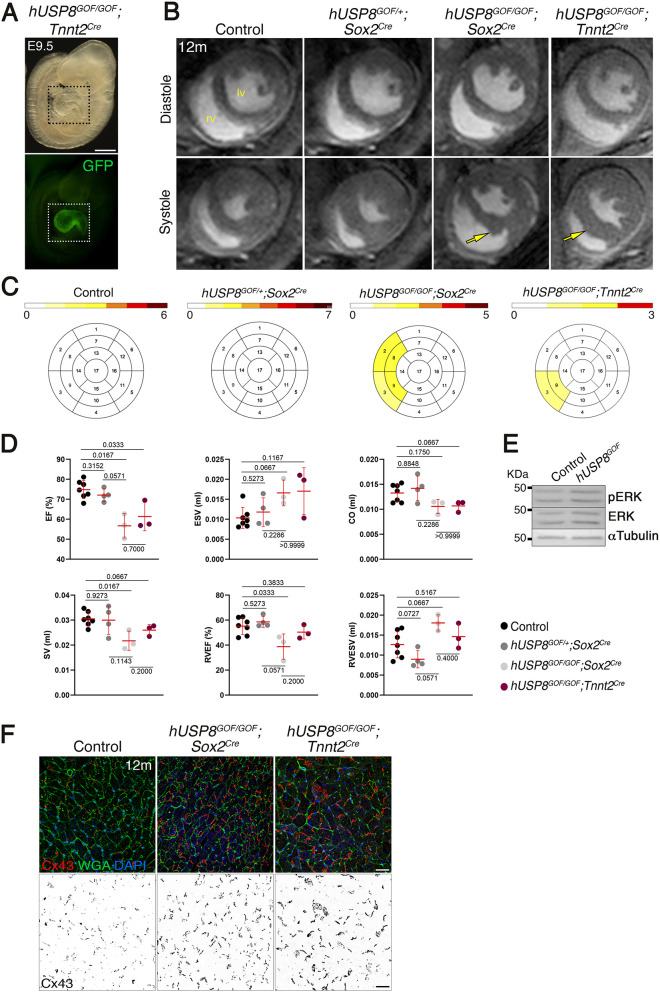
**Myocardial expression of *hUSP8^GOF^* induces cardiac dysfunction.** (A) Bright-field and GFP images of E9.5 control and *hUSP8^GOF/GOF^;Tnnt2^Cre^* embryos. (B) Cardiac magnetic resonance imaging (short axis) from 1-year-old control, *hUSP8^GOF/+^;Sox2^Cre^*, *hUSP8^GOF/GOF^;Sox2^Cre^* and *hUSP8^GOF/GOF^;Tnnt2^Cre^* mice. Arrows indicate septal protrusions. (C) Quantification of septal anomalies using the American Heart Association (AHA) 17-segment model; bull's eye plots show color-coded anomaly scores per group. (D) Cardiac function analysis shows changes in ejection fraction (EF), end-systolic volume (ESV), cardiac output (CO), stroke volume (SV), right ventricular ejection fraction (RVEF) and right ventricular end-systolic volume (RVESV). Graphs show mean±s.d. Data were analyzed using the two-tailed Mann–Whitney test. *n*=3–7 biologically independent mice per genotype. Each data point represents one biological replicate (one mouse). (E) Representative western blot of pERK of controls and *hUSP8^GOF/GOF^;Sox2^Cre^* hearts. (F) Top row: immunofluorescent staining of WGA (green), CX43 (red) and DAPI (blue) in 1-year-old heart sections. Bottom row: thresholded CX43 immunostaining corresponding to the images shown above. Scale bars: 20 μm.

To analyze the specific effect of *hUSP8^GOF^* on cardiac structure and function *in vivo*, we performed cardiac magnetic resonance imaging (MRI) in 1-year-old *hUSP8^GOF/GOF^;Sox2^Cre^* and *hUSP8^GOF/GOF^;Tnnt2^Cre^* mice. Cardiac short-axis images revealed normal overall anatomy of both left (LV) and right (RV) ventricles during systole and diastole across genotypes (Fig. 6B). However, long-axis images showed septal anomalies in both *hUSP8^GOF/GOF^;Sox2^Cre^* and *hUSP8^GOF/GOF^;Tnnt2^Cre^* mice (Fig. 6B, arrows). To quantify these defects, we applied the standardized 17-segment LV myocardial model proposed by the American Heart Association (AHA) (Cerqueira et al., 2002). This analysis identified structural anomalies in segments 2, 3, 8 and 9, corresponding to septal regions, in two of five *hUSP8^GOF/GOF^;Sox2^Cre^* and one of three *hUSP8^GOF/GOF^;Tnnt2^Cre^* hearts (Fig. 6C). We further assessed cardiac function by MRI. Ejection fraction (EF), the percentage of blood pumped out of a filled ventricle with each heartbeat, was significantly reduced in *hUSP8^GOF/GOF^;Sox2^Cre^* (∼57%) and *hUSP8^GOF/GOF^;Tnnt2^Cre^* (∼61%) mice compared to that in heterozygous *hUSP8^GOF/+^;Sox2^Cre^* (∼72%) and control (∼75%) mice (Fig. 6D). End-systolic volume (ESV), the volume of blood remaining in the ventricle after systole, was increased by ∼60% in both homozygous lines, indicating impaired systolic function. LV performance was markedly reduced, with cardiac output (CO), the volume of blood pumped by the LV per minute, decreased by ∼20% in both *hUSP8^GOF^* models (Fig. 6D). Stroke volume (SV), the amount of blood pumped per beat, was reduced by 30% in *hUSP8^GOF/GOF^;Sox2^Cre^* and by 15% in *hUSP8^GOF/GOF^;Tnnt2^Cre^* mice. RV function was also compromised in *hUSP8^GOF/GOF^;Sox2^Cre^* mice, which showed a 25% decrease in RV ejection fraction (RVEF), and a 42% increase in RV end-systolic volume (RVESV), compared to that in controls (Fig. 6D). Diastolic function parameters were comparable across genotypes (Fig. S6). In contrast to skeletal muscle (Fig. 2F), ERK phosphorylation in the myocardium appeared to be unchanged upon hUSP8 activation (Fig. 6E), suggesting a more limited or context-dependent effect in cardiac tissue.

As previously noted, CX43 is a direct target of USP8 (Sun et al., 2018). Consistent with findings in the lens, CX43 signal intensity was increased in both transgenic lines, with a shift from the punctate pattern observed in controls to a more uniform distribution along the plasma membrane in *Sox2^Cre^*- and *Tnnt2^Cre^*-driven transgenic expression in the myocardium (Fig. 6F). Overall, these data suggest that constitutive *hUSP8^GOF^* myocardial expression from early developmental stages is associated with septal defects and reduced systolic function, supporting a role for *hUSP8^GOF^* in myocardial homeostasis.

## DISCUSSION

Mutations in *USP8* are detected in 36.5% of patients with CD, with the *USP8^S718del^* variant being the most common, accounting for 9% of cases (Sbiera et al., 2019). With the aim of generating a preclinical model of genetic CD, we generated a conditional transgenic mouse model expressing the *hUSP8^S718del^* mutation. Using conditional, tissue-specific activation of the transgene, we induced *hUSP8^S718del^* expression throughout epiblast-derived tissues using *Sox2^Cre^*. Surprisingly, transgenic mice did not develop pituitary adenomas. A plausible explanation is that although USP8 is structurally conserved between humans and mouse, its functional interaction with key proteins (EGFR, 14-3-3, etc.) may differ between species; also, epigenetic and microenvironmental conditions (local immunity or paracrine factors) may account for the development of pituitary adenomas. Despite the absence of pituitary adenomas and MAPK signaling overactivation or increased proliferation, transgenic mice showed diffuse corticotroph hyperplasia of ACTH^+^ cells that was not sufficient to overstimulate activity of the HPA axis. This was consistent with the absence of classical CS symptoms (hypercortisolemia, metabolic syndrome, obesity, adrenocortical tumors, or chronic lung and kidney disease; Bertagna et al., 2009). Achieving marked elevations in corticosterone levels in mice requires a continuous, relatively high dose sustained over several days (Dunbar et al., 2010). Moreover, a gradual development of moderate adrenal insensitivity to ACTH was observed during chronic stress in mice, which facilitates HPA axis adaptation to repeated homotypic stressors (Langgartner et al., 2020). Taking these results together, we conclude that the moderate increase in ACTH does not overstimulate the HPA axis; therefore, this transgenic mouse line does not develop CD.

Despite the normal HPA axis function, *hUSP8^GOF^* transgenic mice developed corneal opacities from juvenile stages, accompanied by signs of ongoing wound healing and increased NOTCH signaling activity in the corneal epithelium, a pathway essential for corneal maintenance and repair (Vauclair et al., 2007). Cataracts and corneal abnormalities were associated with membrane defects and mislocalized CX43, a gap junction protein implicated in lens transparency (Gong et al., 2021) and corneal integrity (Grupcheva et al., 2012; Qin et al., 2019; Zhai et al., 2014). Because USP8 directly stabilizes N1ICD (Shin et al., 2020) and regulates CX43 trafficking (Sun et al., 2018), we propose that *hUSP8^GOF^* drives persistent corneal wound healing signaling and disrupts lens remodeling, leading to complex ocular pathologies. Of note, subcapsular cataracts have been reported in patients with CD (Liu et al., 2017; Sharon et al., 2025), and CD in dogs is linked to ocular abnormalities, including corneal defects (Feldman and Nelson, 2004; Laus et al., 2002).

Aged *hUSP8^GOF^* mice developed musculoskeletal abnormalities despite exhibiting normal HPA axis activity. The muscle atrophy observed in these transgenic mice is consistent with the established role of the ubiquitin–proteasome system (UPS) in maintaining skeletal muscle homeostasis (Bodine et al., 2001; Hunt et al., 2021), as well as with the reported increase in pERK signaling (Gallot et al., 2019; Miyake et al., 2017). In addition, USP8 has been shown to modulate WNT signaling in osteoclasts, likely through the regulation of Frizzled receptor stability via deubiquitylation (Chaugule et al., 2021; Mukai et al., 2010). Notably, a human patient carrying a germline *USP8^GOF^* mutation also developed musculoskeletal defects (Cohen et al., 2019), suggesting that the impact of *USP8^GOF^* on musculoskeletal tissues is conserved across humans and mice. Further studies will be necessary to elucidate the molecular and cellular mechanisms driving these cortisol-independent skeletal abnormalities in USP8-associated disease.

Expression of *hUSP8^GOF^* specifically in the myocardium from embryonic stages (via *Tnnt2^Cre^*) led to mild, but significant, reduction in EF, along with septal defects, like those observed in mice with ubiquitous *hUSP8^GOF^* expression. The fact that restricting transgene expression to the myocardium recapitulates the cardiac phenotype observed in *hUSP8^S718del^* ubiquitous models suggests that *USP8^GOF^* has a direct role in the myocardium. Mechanistically, USP8 is known to regulate K63-linked deubiquitylation (Alexopoulou et al., 2016; Sun et al., 2018) and autophagy (Peng et al., 2020), a tightly controlled process involved in multiple forms of cardiomyopathy (Calise and Powell, 2013; Chen et al., 2016; Zheng et al., 2011). In addition, the altered localization of CX43 in the myocardium could disrupt gap junction structure and function, thereby compromising cardiac performance (Araujo et al., 2025). Notably, reduced EF has also been reported in a patient carrying a germline *USP8^GOF^* mutation (Cohen et al., 2019), leading us to speculate that myocardial expression of *hUSP8^GOF^* may contribute to cardiac comorbidities in these patients, alongside the effects of elevated cortisol levels.

To date, two germline variants of *USP8* have been described in humans, both associated with multiorgan abnormalities (Cohen et al., 2019; Sakamoto et al., 2024). However, only the patient carrying a *USP8^GOF^* mutation affecting the same 14-3-3 binding domain as in our transgenic model developed CD (Cohen et al., 2019). The similarities between this patient and our mouse model point to a conserved role of USP8 in maintaining the homeostasis of cardiac, musculoskeletal and ocular tissues. Overall, this new conditional transgenic mouse line expressing the *hUSP8^S718del^* mutation highlights the importance of UPS regulation in multiorgan homeostasis and uncovers additional roles for this pathogenic *USP8^S718del^* variant.

A limitation of this study is that the *hUSP8^S718del^;Sox2^Cre^* model does not fully reproduce the endocrine manifestations of CD, particularly ACTH-secreting pituitary adenomas and sustained hypercortisolemia. Although transgenic mice developed diffuse corticotroph hyperplasia with moderately increased ACTH levels, HPA axis activity remained largely preserved. These findings suggest that USP8^GOF^ alone may be insufficient to drive full pituitary tumorigenesis in mice and that additional species-specific, genetic, epigenetic or microenvironmental factors may contribute to disease development in humans. We were also unable to consistently detect the cleaved ∼90 kDa active form of hUSP8 in either control or transgenic tissues, which may reflect technical limitations of the chosen antibody. Finally, although the phenotypes observed are consistent with enhanced USP8 activity, we did not perform direct cell-based deubiquitinase assays to quantify USP8 overactivation. Future studies combining biochemical and cell-based functional approaches will be important to further define the molecular mechanisms underlying tissue-specific USP8 GOF pathology.

## MATERIALS AND METHODS

### Mice

Mouse studies were approved by the CNIC Animal Experimentation Ethics Committee and by the Community of Madrid (PROEX 054.6/25). All animal procedures conformed to European Union (EU) Directive 2010/63/EU and Recommendation 2007/526/EC on the protection of animals used for experimental and other scientific purposes, enacted in Spanish law under Real Decreto 118/2021 (modification on Real Decreto 53/2013) and Ley 32/2007. Mice were housed in specific pathogen-free conditions at the CNIC Animal Facility, under a temperature-controlled environment of 21-23°C within a 12-h light/12-h dark cycle and *ad libitum* food and water access. Mice were maintained in a C57BL/6 inbred background.

### Generation of a conditional *hUSP8^GOF^* mouse line

Site-directed mutagenesis of *FLAG-HA-hUSP8* (Addgene plasmid #22608) was performed using the QuikChange II XL kit with PfuUltra polymerase and primers introducing the p.S718del mutation (Table S2). After *Dpn*I digestion, products were transformed into *Escherichia coli* DH5α. Plasmid DNA was purified and verified by Sanger sequencing (Eurofins) (Table S2). *Nhe*I sites were added to both ends of the *FLAG-HA-hUSP8^S718del^* cDNA via PCR with Phusion polymerase and specific primers (Table S2). The PCR product was *Nhe*I digested and cloned into the *Nhe*I site of the laboratory-modified *pBigT-IRES-eGFP* vector (Addgene plasmid #21270), which contains a *loxP*-flanked *PGK-NeoR* cassette, *IRES* and *eGFP*. Cloning occurred between the *polyA STOP* and *IRES* sequences (Fig. S1A), and insert orientation was confirmed by *Nde*I/*Xma*I digestion. The resulting *FLAG-HA-hUSP8^S718del^-IRES-eGFP* construct was digested with *Pac*I/*Asc*I and inserted into the *Pac*I/*Asc*I sites of a modified *pROSA26-1* vector (Soriano, 1999) containing 5.4 kb *Rosa26* homology arms and a *PGK-DTA* cassette (Fig. S1A). GFP expression was confirmed in G4 mouse embryonic stem cells co-transfected with the construct and the Cherry-Cre vector pLM-CMV-R-Cre (Addgene plasmid #27546). The final construct, *pR26-FloxSTOPFlox-FLAG-HA-hUSP8S718del::IRES-GFP*, was linearized with *Pvu*I, and the appropriate fragment was electroporated into mouse embryonic stem cells (129X1/Bl6 background). After 7 days of G418 selection (200 μg/ml), 125 clones were screened. Southern blot analysis using 5′ and 3′ probes confirmed eight positive clones (Fig. S1B), and three were selected for karyotyping. One confirmed clone was injected into C57BL/6 blastocysts to generate chimeras. Germline transmission was verified by PCR genotyping (Table S1), and a single male founder was used to establish the colony. Mice were genotyped using the primers in Table S2.

The *Sox2^Cre^* and *Tnnt2^Cre^* mice were genotyped as described (Hayashi et al., 2002b; Jiao et al., 2003).

Mice were euthanized in a carbon dioxide chamber and weighed before tissue sample collection. CD is threefold to fivefold more prevalent in women than in men (Bertagna et al., 2009; Castinetti et al., 2012; Clayton et al., 2016), and CD pathophysiology exhibits sex differences (Pecori Giraldi et al., 2020). We showed only female data in canonical figures for clarity; however, our study examined male and female mice, and similar results were found for both sexes.

### Bright-field microscopy and confocal imaging

Whole-mount and bright-field images were captured using Leica MZFL III or Olympus BX51 microscopes with DP71 CCD cameras and cellSens software. Confocal immunofluorescence images were acquired on a Leica SP8 using LAS X 3.5.7. Images were processed and analyzed with ImageJ Fiji, NDP.view2 or Adobe Photoshop.

### Western blotting

Protein was extracted at 4°C using SDS lysis buffer with inhibitors, followed by tissue homogenization with a cordless grinder and sonication (Bioruptor UCD-200, ≥5 min). For anti-HA blots, S-100 CHAPS buffer was used. Lysates were centrifuged (18,440 ***g***, 15 min, 4°C), and supernatants were stored at −80°C. Protein concentration was measured with the Pierce BCA assay (Thermo Fisher Scientific). At least 20 μg protein was denatured in Laemmli buffer at 95°C for 5 min and separated on 10% SDS-PAGE gels. Proteins were transferred to methanol-activated PVDF membranes (0.45 μm) and blocked in 5% bovine serum albumin (BSA) or 5% milk (anti-HA) in Tris-buffered saline 0.1% Tween-20 (TBST). Primary antibodies were incubated overnight at 4°C; secondary antibodies were incubated for 1 h at room temperature (RT). Detection was done using horseradish peroxidase (HRP) substrates (Immobilon Classico, Crescendo or Forte). Images were acquired with an iBright CL1500 (Thermo Fisher Scientific) or developed on film using a CURIX 60 (AGFA) and scanned on an MP C4503PS (RICOH). The antibodies used are listed in Table S3.

### RT-qPCR

Total RNA was extracted using an RNeasy Kit (QIAGEN) according to the manufacturer's instructions. Complementary DNA (cDNA) was generated from 1 μg total RNA using a High-Capacity cDNA Reverse Transcription Kit (Thermo Fisher Scientific) with random hexamers. RT-qPCR amplification of cDNA was performed with Power SYBR Green PCR Mastermix 2X (Thermo Fisher Scientific) on a QuantStudio 5 Real-Time PCR System (Thermo Fisher Scientific) programmed with standard cycling parameters. Relative expression levels of the genes of interest were calculated using the 2^−ΔΔCT^ method described in Livak and Schmittgen (2001) after normalizing to that of *Gapdh* using the following primers: *Gapdh* forward, 5′-AACTTTGGCATTGTGGAAGG-3′, reverse, 5′-ACACATTGGGGGTAGGAACA-3′; *eGFP* forward, 5′-ACGTAAACGGCCACAAGTTC-3′, reverse, 5′-ACGTAGCCTTCGGGCATG-3′; *FLAG-HA* forward, 5′-GACTACAAGGATGACGATGACAA-3′, reverse, 5′-GATAACCTCGAGCAGCGTAAT-3′; *hUSP8* (Rong et al., 2020) forward, 5′-CTGCTGTGGCTTCAGTTCCT-3′, reverse, 5′-GGAAATAATCCTGCTGTTGCTTGA-3′; *mUsp8* forward, 5′-CGAGAGCACGAAGTGAAGAA*-*3′, reverse, 5′-GGGAATGGTAGTAACGAAAGGT-3′; *Pomc* forward, 5′-ATGCCGAGATTCTGCTACAGT-3′, reverse, 5′-TCCAGCGAGAGGTCGAGTTT-3′.

### Plasma and urinary biochemical analysis

Blood and urine samples were collected simultaneously. Blood (150 μl) was obtained biweekly via submandibular puncture into heparin tubes. Plasma was separated by centrifugation (850 ***g***, 15 min, within 2 h), and hemolysis was visually scored (0–3); only samples with slight or no hemolysis were stored at −80°C. Plasma levels of creatine kinase, alkaline phosphatase, alanine transferase, free and total cholesterol, creatinine, glucose, triglycerides and urea were measured using a Dimension RxL Max analyzer (Siemens). Urine was collected hourly from 07:00 to 15:00 using a stress-minimizing method: mice were placed in a sterile hood, and urine was collected with sterile tips into tubes. At least 300 μl was obtained per animal, centrifuged (100 ***g***, 10 min) and stored at −80°C. Urinary creatinine and free cortisol in mice were analyzed by Laboratorios Echevarne (Madrid) using chemiluminescence and UV-Vis spectrophotometry.

### Histological procedures

Thoracic organs were exposed by cutting the ribcage, and, after resecting the descending aorta, the heart was perfused via the right ventricle with 5 ml PBS and 1 ml 4% paraformaldehyde (PFA). The heart was then isolated and weighed. Eyes, lungs, kidneys, adrenal glands, pituitary glands and lateral gastrocnemius muscles were collected following standard protocols (Cao et al., 2017; Ionescu et al., 2016; Kittel et al., 2004; Morawietz et al., 2004). Tissues were fixed for 48 h at 4°C in 4% PFA (eyes in Davidson's fixative; Moore and Barr, 1954), then washed in 70% ethanol overnight at 4°C. Paraffin embedding and orientation were performed at the CNIC Histopathology Unit following Registry of Industrial Toxicology Animal-data (RITA) and North American Control Animal Database (NACAD) guidelines (Kittel et al., 2004; Morawietz et al., 2004). Tissues were sectioned at 4 μm using a semi-automated rotary microtome and stored at 4°C. Hematoxylin and Eosin (H&E) and Picro Sirius Red staining was done using standard protocols. At least three samples per genotype were analyzed, and representative images were selected.

### Immunofluorescence on sections

4-μm paraffin sections were washed twice for 5 min with xylene and rehydrated by a series of ethanol solutions of decreasing concentrations (2×100%, 95%, 90%, 70%, 50%, 3 min each), followed by Milli-Q water washes. For antigens that required unmasking (N1ICD, Ki67, CX43), antigen retrieval was performed by boiling the sections in 10 mM sodium citrate buffer pH 6 for 10 min. After boiling, sections were cooled down to RT in the solution for 20 min. Subsequently, endogenous peroxidases were quenched with 1% hydrogen peroxide solution in methanol for 30 min. Tissues were permeabilized with PBS-0.3% Triton X-100. Blocking was done for 1 h at RT with regular blocking solution [5% heat-inactivated fetal bovine serum (FBS) in PBS] or histoblock solution (3% BSA, 5% FBS, 20 mM MgCl_2_ and 0.3% Tween-20). The primary antibodies were diluted in blocking solution, and sections were incubated overnight at 4°C. For antigens that did not require amplification, fluorescent dye-conjugated secondary antibodies were used in the blocking solution for 1 h at room temperature. For antigen signal amplification, a secondary biotin-conjugated antibody was used, followed by the formation of avidin–biotinylated HRP complexes using a VECTASTAIN Elite ABC kit (Vector Laboratories, PK-6100). Alternatively, a secondary HRP-conjugated antibody was used. Signal detection was achieved using Tyramide Signal Amplification Plus Fluorescein (PerkinElmer, NEL741B001KT) at RT according to the manufacturer's instructions. Sections were washed in PBS before incubation with DAPI and other secondary antibodies for 1 h at room temperature. The slides were washed in Milli-Q water and mounted with Fluoromount. The antibodies and dyes used are listed in Table S3.

### Quantification of ACTH^+^ cells in pituitary sections

Tile scan pictures were taken from whole pituitaries. Insets were taken to a field of 33,457.033 μm^2^ in the adenohypophysis region. The total number of DAPI^+^ nuclei was counted using the StarDist2 Plugin from ImageJ Fiji (Schmidt et al., 2018), and the number of ACTH^+^ cells per cluster was manually counted. The ratio of total ACTH/DAPI cells and the average number of ACTH^+^ cells per cluster were represented. At least six representative field pictures from three non-consecutive sections per biological sample were analyzed.

### Quantification of cellular proliferation

Cell proliferation was assessed by Ki67 immunofluorescence. Insets were taken to a field of 33,457.033 μm^2^ in the adenohypophysis region. DAPI^+^ total nuclei were quantified using the StarDist2 Plugin from ImageJ Fiji (Schmidt et al., 2018), followed by manual annotation of Ki67^+^ cells. At least six representative field pictures from three non-consecutive sections per biological sample were analyzed.

### Quantification of cross-sectional area in skeletal muscle sections

Picro Sirius Red staining was used to label skeletal muscle cell membranes. Images were captured from different territories of the lateral gastrocnemius. Area quantification was performed using the ImageJ Fiji freehand tool, and the cross-sectional area was manually delineated. Area measurements were averaged for each biological replicate. At least four representative images and 20 cells per image were analyzed per biological sample.

### Assessment of corneal opacification and cataract severity

Corneal opacification was scored as present or absent based on signs such as epithelial thickening, keratinization, stromal fibrosis with vascularization, or keratitis. Cataract severity was classified as mild (subtle fiber disarrangement/swelling, slight vacuolization, few retained nuclei), moderate (clear fiber degeneration, nuclear retention, few deposits) or severe (pronounced degeneration, extensive nuclear retention, large deposits, capsular enlargement, lens bulging).

### Quantification of retinal inner nuclear layer

The inner nuclear area fraction (INL) was calculated as (area occupied by INL nuclei/total INL area)×100. We used three non-consecutive retinal sections from three biological replicates. For quantification, we used ImageJ Fiji. Briefly, the total INL area was manually delineated and measured. Color deconvolution was performed using the H&E vector to isolate the Hematoxylin channel. A binary mask was then generated by thresholding the channel to accurately identify INL nuclei. Lastly, the area occupied by INL nuclei was measured from this mask, and the percentage of occupation was calculated.

### *In vivo* image acquisition

The imaging study was conducted on four groups of mice from two mouse lines according to the Cre line: controls (*hUSP8^GOF/+^;Sox2^+/+^*, *hUSP8^GOF/GOF^;Sox2^+/+^*, *hUSP8^GOF/+^;Tnnt2^+/+^* or *hUSP8^GOF/GOF^;Tnnt2^+/+^*), transgenic heterozygous (*hUSP8^GOF/+^;Sox2^Cre^*) and transgenic homozygous (*hUSP8^GOF/GOF^;Sox2^Cre^* or *hUSP8^GOF/GOF^;Tnnt2^Cre^*).

At 12 months, MRI, PET and CT imaging were used to assess cardiac function, structural adipose tissue, glucose metabolism and skeletal structures. MRI distinguishes fat and water protons (Marzola et al., 2016). Besides analyzing bone morphology, PET/CT allows measurement of uptake of the glucose analog radiotracer 2-deoxy-2-[^18^F]fluoro-D-glucose (^18^F-FDG) in brain, liver and heart (de Francisco et al., 2021). Each group included at least three animals. Mice were anesthetized with 2% isoflurane (1.8 liters/min O_2_) and protected with ophthalmic gel during imaging.

MRI was performed on a 7-T Agilent/Varian scanner using an electrocardiogram-triggered fast gradient echo cine sequence with the parameters: repetition time (TR)/echo time (TE) ∼125/1.25 ms; 30×30 mm field of view; 128×128 acquisition matrix; 15° flip angle; four averages; 20 cardiac phases; ∼12 slices (0.8 mm thick, 0.2 mm gap). Orthogonal chest images guided acquisition of long- and short-axis heart views to ensure complete LV coverage. Cardiac MRI was performed using an actively shielded 115/60 gradient. PET/CT was conducted using a small-animal scanner. Mice received 14.6±1.3 MBq ^18^F-FDG intravenous administration, and PET data were collected over 30 min and reconstructed with Teratomo 3D (six subsets, four iterations, 85×65×236 matrix, voxel size 0.4 mm). CT was acquired post-PET with 178 μA current, 45 kVp tube voltage, 360 projections (500 ms, pitch 1.5, binning 1:4) and reconstructed using the Ramlack algorithm.

### *In vivo* image quantification

Cine MRI data were analyzed using Segment v1.9 R3819. Endocardial and epicardial contours were manually traced on short-axis slices to calculate ventricular end-diastolic volume (EDV), ESV, SV, EF and mass (VM) (Hernandez-Porras et al., 2014). Structural abnormalities in the LV, such as trabeculations, were assessed by an expert using the 17-segment AHA model (Cerqueira et al., 2002). The LV was divided into basal (segments 1–6), mid (7–12), apical (13–16) and apex (17). Each segment was scored (0, normal; 1, abnormal), and results were summarized in a bull's-eye plot by coronary territory. PET/CT images were analyzed using a Multimodality Workstation (Desco et al., 1999). For PET, regions of interest were drawn in the brain, heart, and liver to obtain mean and maximum standardized uptake values. CT-based skull measurements (length, width, intercanthal distance) were taken from coronal and sagittal maximum-intensity projections (MIPs). Kyphosis angles were assessed on whole-body sagittal MIPs. Right femur bone density was estimated by HU after manual segmentation in 3D Slicer.

### Statistics and data representation

Statistical analyses and graphical representations were performed using GraphPad Prism 10.4.0. All experiments were carried out with at least three biological replicates. First, data normality was assessed using the Shapiro–Wilk test. Normally distributed data were analyzed by performing unpaired two-tailed *t*-tests with Welch's correction for significance, with a confidence level of 95%. When normality was not supported, the Mann–Whitney *U*-test was performed. Differences were considered statistically significant at *P*<0.05. Data and error bars are presented as mean±s.d. *P*-values are indicated in figures. Mice were genotyped before the experiment, and the investigators were aware of experimental groups.

## Supplementary Material

10.1242/dmm.052695_sup1 Supplementary information

Table S1. Primers used for the generation of the conditional hUSP8GOF mouse line.
