# Supplementary material for: Systemic and cardiac pathology induced by a clinically relevant USP8 activating mutation
Source: Dis Model Mech. 2026 Jul 22;19(7):dmm052695. doi: 10.1242/dmm.052695 (PMC13446564; doi:10.1242/dmm.052695)
Supplement: Supplementary file 1 [file dmm-19-052695-s1.pdf]

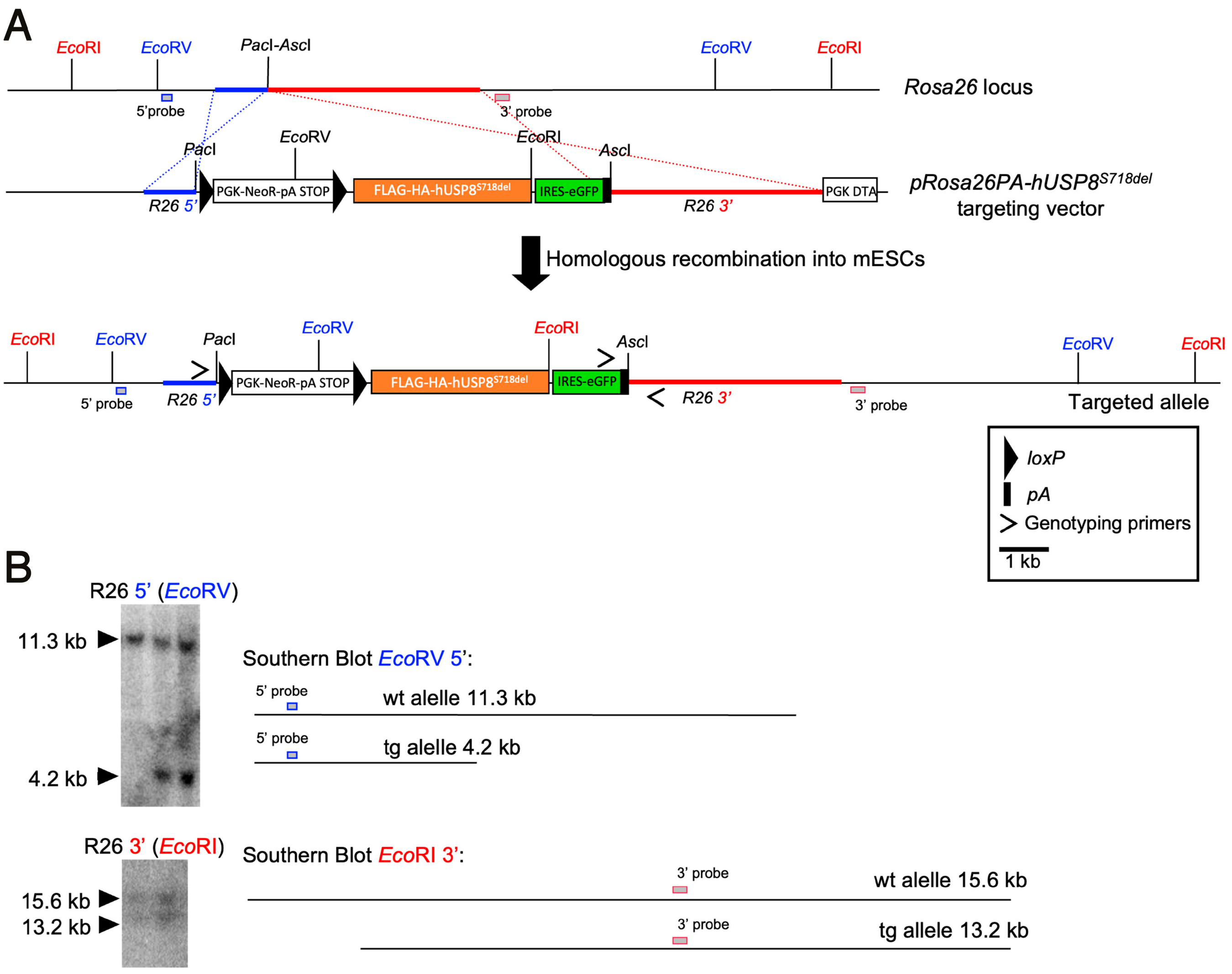

Fig. S1. Targeting strategy for *hUSP8^{GOF}* allele. (A) Schematic of *Rosa26*-targeted insertion in mESCs: a floxed *PGK-NeoR-pA STOP* cassette, *hUSP8^{S718del}* cDNA (FLAG-HA tagged), and *IRES-eGFP* inserted by homologous recombination. Top: *Rosa26* wild-type locus with 5′ and 3′ Southern probes. Middle: targeting vector with *loxP* sites, *pA* signal (black squares), and genotyping primers (black arrowheads). Bottom: targeted *hUSP8^{GOF}* allele. Scale bar: 1 kb. Abbreviations: mESCs, mouse embryonic stem cells; pA, polyadenylation signal; NeoR, neomycin resistance; GFP, green fluorescent protein; PGK, phosphoglycerate kinase; DTA, diphtheria toxin; IRES, internal ribosome entry site. **(B)** Southern blot of EcoRV- and EcoRI-digested genomic DNA from wt and transgenic mESC clones, showing 4.2 kb and 13.2 kb bands with 5′ and 3′ probes, respectively.

Disease Models & Mechanisms • Supplementary information

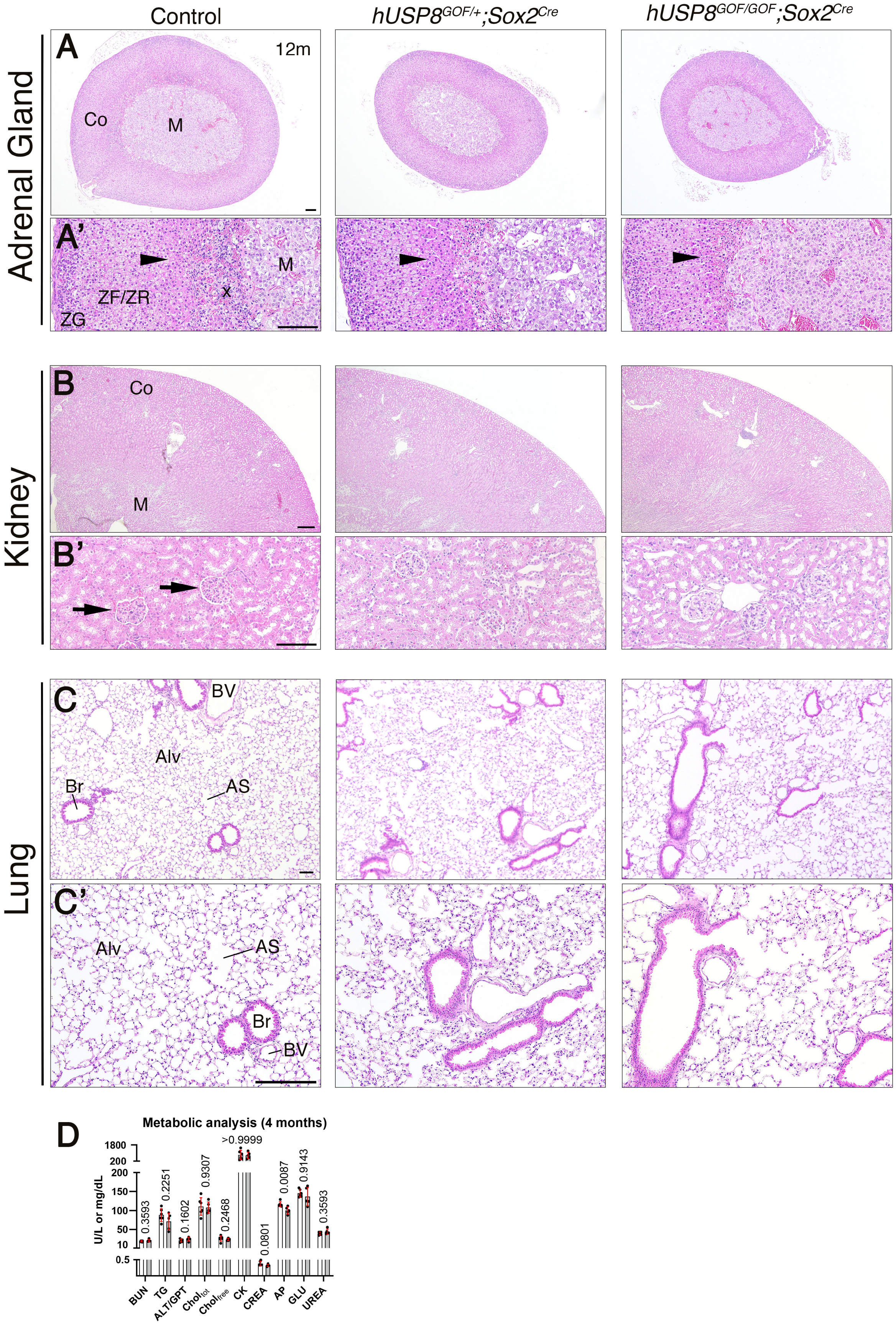

Disease Models & Mechanisms • Supplementary information

Fig. S2. Normal adrenal, kidney, and lung histology in *hUSP8$^{GOF/GOF}$;Sox2^{Cre}* mice. (A-C) H&E-stained longitudinal sections of adrenal gland (**A**), kidney (**B**), and lung (**C**) from 1-year-old control, *hUSP8$^{GOF/+}$;Sox2^{Cre}* and *hUSP8$^{GOF/GOF}$;Sox2^{Cre}* 1-year-old mice. **(A'-C')** Higher magnification. **(A, A')** Adrenal cortex (Co) and medulla (M) with zona glomerulosa (ZG), fasciculata (ZF), reticularis (ZR), and X-zone (X); arrowheads mark reticular cells. **(B, B')** Kidney cortex (Co) and medulla (M); arrows point to glomeruli. **(C, C')** Lung parenchyma with alveoli (Alv), alveolar sacs (AS), terminal bronchiole (Br), and blood vessels (BV). (**D**) Blood plasma metabolic panel at 4-months: BUN: blood urea nitrogen; TG: triglycerides; ALT/GPT: alanine amino transferase/glutamic-pyruvic transaminase; $Chol_{tot}$: total cholesterol; $Chol_{free}$: free cholesterol; CK: creatine kinase; CREA: creatinine; AP: alkaline phosphatase; Glu: glucose; Urea (calculated as BUN*2.14). Enzyme activity was measured in units (U) per L, and metabolites concentration in mg per dL. Bars represent means values ± S.D. Mann-Whitney U test was performed. *P* values are indicated. Scale bars: 100 µm.

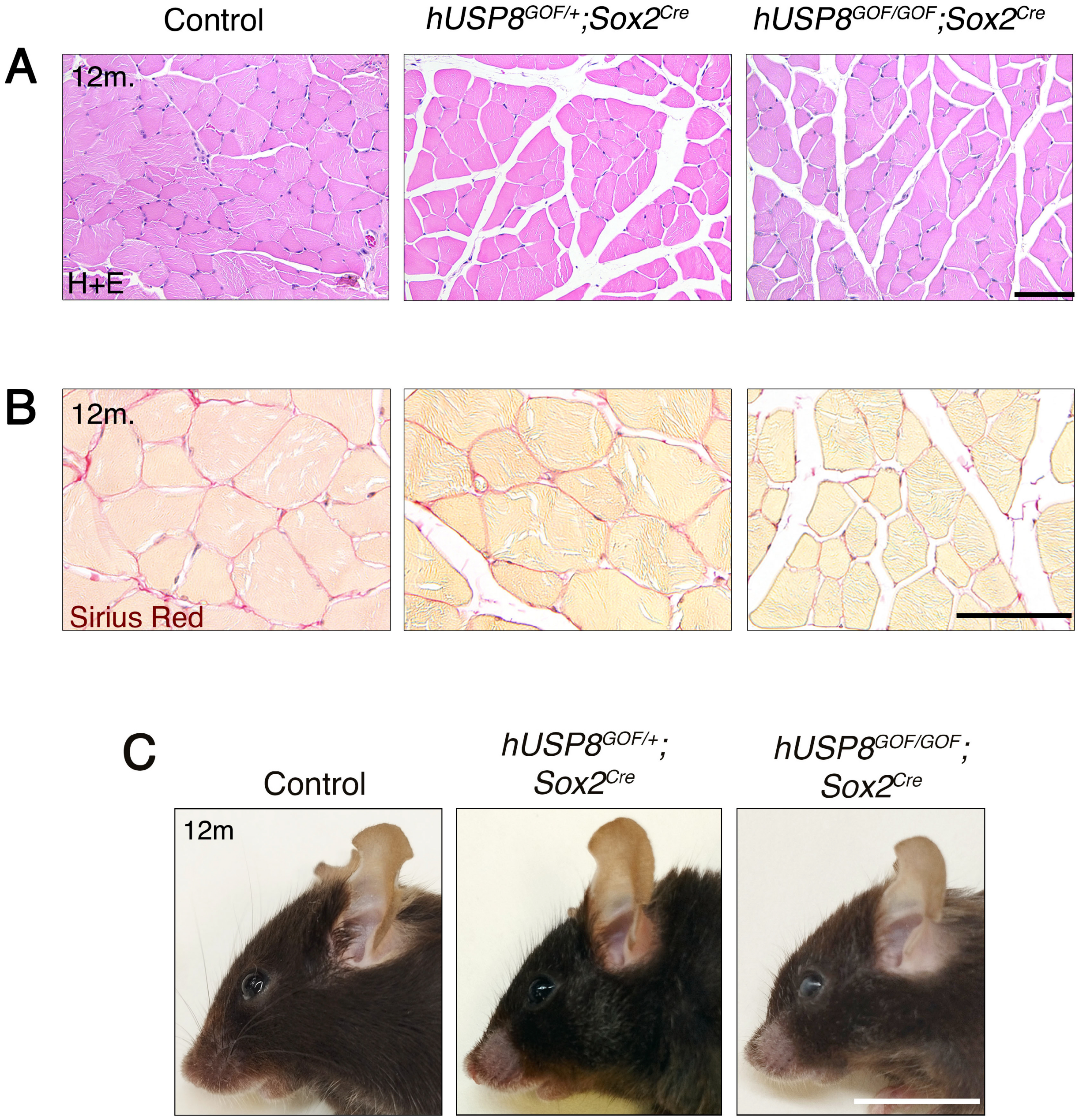

Fig. S3. Muscular and skeletal defects in *hUSP8*$^{GOF/GOF}$*;Sox2*^{Cre} mice. (**A**) H&E and (**B**) Picro Sirius Red-stained cross-sections of lateral gastrocnemius muscle from 1-year-old control, *hUSP8*^{GOF}*;Sox2*^{Cre} and *hUSP8*$^{GOF/GOF}$*;Sox2*^{Cre} mice. **(C)** Lateral view showing head malformation in *hUSP8*$^{GOF/GOF}$*;Sox2*^{Cre} mice. Scale bars: A, B: 100 µm; C: 1 cm.

Disease Models & Mechanisms • Supplementary information

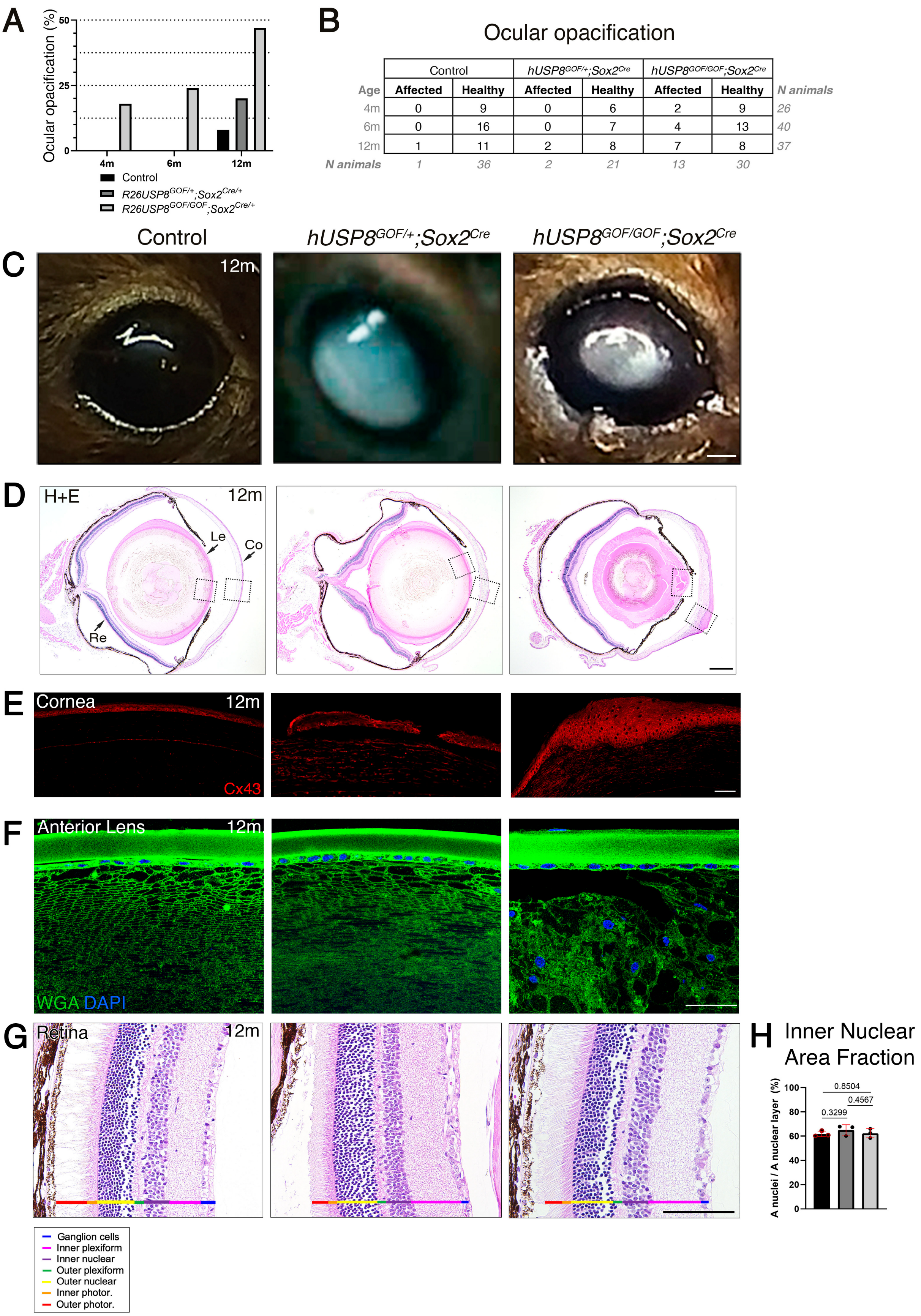

Ocular opacification

Age	Control Affected	Control Healthy	hUSP8GOF/+;Sox2Cre Affected	hUSP8GOF/+;Sox2Cre Healthy	hUSP8GOF/GOF;Sox2Cre Affected	hUSP8GOF/GOF;Sox2Cre Healthy	*N animals*
4m	0	9	0	6	2	9	*26*
6m	0	16	0	7	4	13	*40*
12m	1	11	2	8	7	8	*37*
N animals	*1*	*36*	*2*	*21*	*13*	*30*	

Disease Models & Mechanisms • Supplementary information

Fig. S4. Ocular phenotypes in *hUSP8$^{GOF/GOF}$;Sox2^{Cre}* mice. (**A**) O cular opacification incidence at 3, 6, and 12 months. (**B**) Frequency table of affected mice. (**C**) Visible opacification in 1-year-old transgenic eyes. (**D**) H&E of sagittal eye sections from 1-year-old control, *hUSP8$^{GOF/+}$;Sox2^{Cre}* and *hUSP8$^{GOF/GOF}$;Sox2^{Cre}* mice. Le, lens; Co, cornea; Re, retina. (**E**) Cx43 staining in corneal sections. (**F**) WGA and DAPI in lens sections showing disrupted membrane organization. (**G**) H&E-stained retina sections. Color-coded bars indicate lens layers. **(H)** Quantification of retina inner nuclear area fraction. Graphs show mean ± S.D.; unpaired two-tailed Welch's t-test. P values are indicated. Scale bars: C, D: 500 µm; E–G: 100 µm.

Disease Models & Mechanisms • Supplementary information

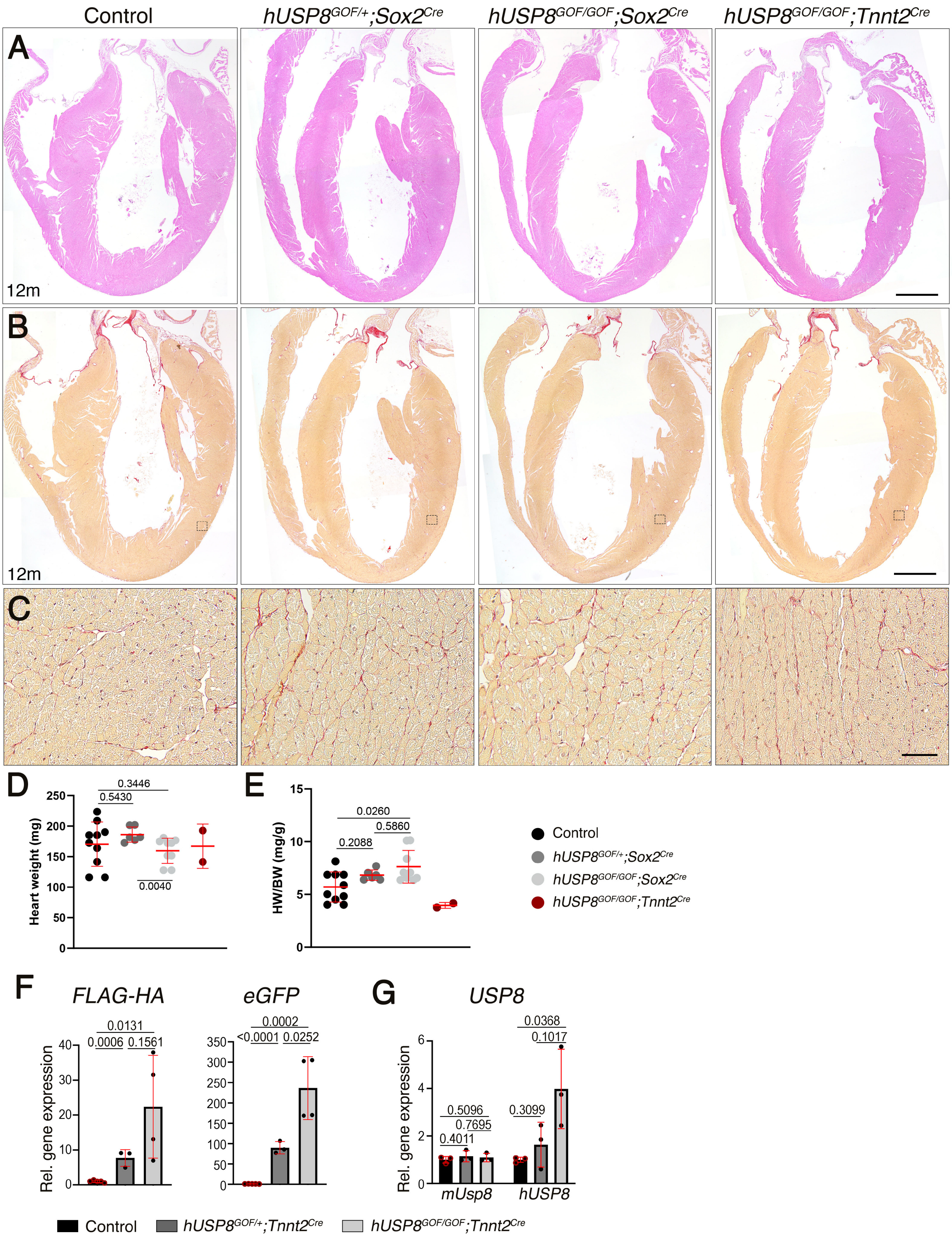

Disease Models & Mechanisms • Supplementary information

Fig. S5. Cardiac histology and morphometry in *hUSP8^{GOF}* mic e. (A) H&E and **(B, C)** Picro Sirius Red stainings of longitudinal heart sections from 1-year-old control, *hUSP8$^{GOF/GOF}$;Sox2^{Cre}*, and *hUSP8$^{GOF/GOF}$;Tnnt2^{Cre}* mice. **(D)** Heart weight and **(E)** heart-to-body weight ratio (HW/BW). Graphs show mean ± S.D.; Mann-Whitney U test was performed. *P* values are indicated. **(F)** RT-qPCR for *FLAG-HA*, *eGFP* and **(G)** mouse and human *USP8* in E11.5 control and *hUSP8$^{GOF/GOF}$;Tnnt2^{Cre}* hearts. *FLAG-HA*, *eGFP* and *USP8* were normalized to *Gapdh* expression; unpaired *t*-test. *P* values are indicated. Scale bars: A, B: 1 mm; C: 50 μm.

Disease Models & Mechanisms • Supplementary information

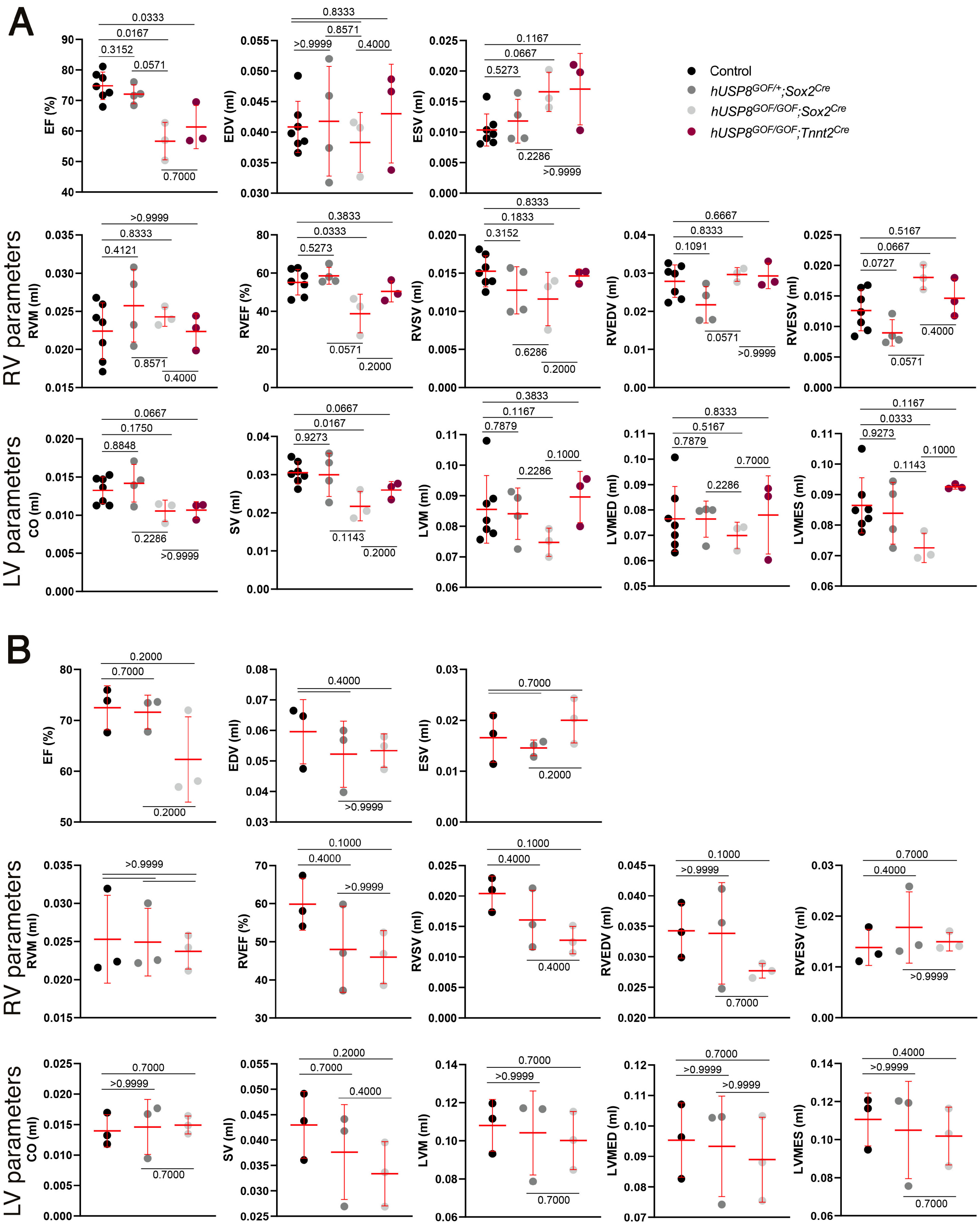
A
EF (%)
0.0333
0.0167
0.3152
0.0571
0.7000
EDV (ml)
0.8333
0.8571
>0.9999
0.4000
ESV (ml)
0.1167
0.0667
0.5273
0.2286
>0.9999
Control
$hUSP8^{GOF/+};Sox2^{Cre}$
$hUSP8^{GOF/GOF};Sox2^{Cre}$
$hUSP8^{GOF/GOF};Tnnt2^{Cre}$
RV parameters
RVM (ml)
>0.9999
0.8333
0.4121
0.8571
0.4000
RVEF (%)
0.3833
0.0333
0.5273
0.0571
0.2000
RVSV (ml)
0.8333
0.1833
0.3152
0.6286
0.2000
RVEDV (ml)
0.6667
0.8333
0.1091
0.0571
>0.9999
RVESV (ml)
0.5167
0.0667
0.0727
0.4000
0.0571
LV parameters
CO (ml)
0.0667
0.1750
0.8848
0.2286
>0.9999
SV (ml)
0.0667
0.0167
0.9273
0.1143
0.2000
LVM (ml)
0.3833
0.1167
0.7879
0.1000
0.2286
LVMED (ml)
0.8333
0.5167
0.7879
0.7000
0.2286
LVMES (ml)
0.1167
0.0333
0.9273
0.1000
0.1143
B
EF (%)
0.2000
0.7000
0.2000
EDV (ml)
0.4000
>0.9999
ESV (ml)
0.7000
0.2000
RV parameters
RVM (ml)
>0.9999
RVEF (%)
0.1000
0.4000
>0.9999
RVSV (ml)
0.1000
0.4000
0.4000
RVEDV (ml)
0.1000
>0.9999
0.7000
RVESV (ml)
0.7000
0.4000
>0.9999
LV parameters
CO (ml)
0.7000
>0.9999
0.7000
SV (ml)
0.2000
0.7000
0.4000
LVM (ml)
0.7000
>0.9999
0.7000
LVMED (ml)
0.7000
>0.9999
>0.9999
LVMES (ml)
0.4000
>0.9999
0.7000

Fig. S6. Sex-specific cardiac function in systemic and cardiac-sp ecific *hUSP8^{GOF}* mice. MRI-based cardiac functional analysis of 1-year-old control, *hUSP8$^{GOF/+}$;Sox2^{Cre}*, *hUSP8$^{GOF/GOF}$; Sox2^{Cre}* and *hUSP8$^{GOF/GOF}$;Tnnt2^{Cre}* mice grouped by sex: **(A)** females and **(B)** males. Parameters include left (LV) and right ventricular (RV) measures: EF, ejection fraction; EDV, end-diastolic volume; ESV, end-systolic volume; RVM, right ventricular (RV) mass; RVEF, RV ejection fraction; RVSV, RV stroke volume; RVEDV, RV end-diastolic volume; RVESV, RV end-systolic volume; CO, cardiac output; SV, stroke volume; LVM, left ventricular (LV) mass; LVMED, LV mass at end-diastole; LVMES, LV mass at end-systolic. Graphs show mean ± S.D.; Mann-Whitney U test was performed. P values are indicated.

Table S1. Primers used for the generation of the conditional *hUSP8^{GOF}* mouse line.

Available for download at
https://journals.biologists.com/dmm/article-lookup/doi/10.1242/dmm.052695#supplementary-data

Disease Models & Mechanisms • Supplementary information

Table S2. Primers used for the generation of the conditional *hUSP8*^{GOF} mouse line.

Primer purpose	Sequence (5′→3′)
Generation of *Ser718* deletion	F: CTGAAGCGCTCCTACTCCCCAGATATAACC<br>R: GGTTATATCTGGGGAGTAGGAGCGCTTCAG
Sequencing of *Ser718* deletion	CCCAATCACTGGAACCTTTCG
Generation of *Nhe*I sites	F:<br>R:
Primers for amplification of *R26* 5′ probe	F: CATACGCCACAGGGAGTCCAA<br>R: CTTCATCTAGTCGACCCCAC
Primers for amplification of *R26* 3′ probe	F: AGGACAGTGCTTGTTTAAGGC<br>R: GTTTTATAATTTGGAACTTTG
Genotyping primers for germline transmission (*R26*	F *R26* 5′: AAAGTCGCTCTGAGTTGTAT<br>F tg: GGAGCGGGAGAAATGGATATG<br>R *R26* 3′: TGCCCGACAACCACTACCTGAG
Genotyping primers for germline	F: CCTGGCAGAAACACAAGCAG<br>R: GCTGAACTTGTGGCCGTTTAC

F, forward; F tg, forward transgene; R, reverse; *R26*, *Rosa26* locus.

Disease Models & Mechanisms • Supplementary information

Table S3. Antibodies and dyes used in this study.

Antibodies and dyes	Company	Catalog#	Dilution
TNNT2	DSHB	CT3	1:50
N1ICD (V1744)	Cell Signaling	4147	1:100
CX43	Sigma-Aldrich	C6219	1:100
ACTH	Ray Biotech	130-10364-20	1:100
Troponin I (H-170)	Santa Cruz	sc-15368	1:100
Ki67 (SolA15)	Thermo Fisher Scientific	14-5698-82	1:100
GFP	Origene	R1091AP	1:100 (WB)
α-Tubulin	Thermo Fisher Scientific	62204	1:30,000 (WB)
Anti-mouse HRP	Dako	0447	1:3000 (WB)
Anti-rabbit HRP	Dako	0448	1:3000 (WB)
Anti-goat HRP	Thermo Fisher Scientific	PA128664	1:3000 (WB)
Biotinylated anti-rabbit	Vector Laboratories	BA-1000	1:200
AF647 anti-ERG	Abcam	ab196149	1:100
AF647 anti-HA	Cell Signaling	2367S	1:1000 (WB)

Disease Models & Mechanisms • Supplementary information

Antibodies and dyes	Company	Catalog#	Dilution
AF488 anti-rabbit	Thermo Fisher Scientific	A21206	1:200
AF568 anti-rabbit	Thermo Fisher Scientific	A10042	1:200
AF568 anti-mouse	Thermo Fisher Scientific	A10037	1:200
AF488 anti-mouse	Thermo Fisher Scientific	A11029	1:200
WGA-AF488	Cell Signaling	7077	1:100
USP8 (E-1)	Santa Cruz Biotechnology	sc-376130	1:200 (WB)
Total ERK1/2	Cell Signaling	9102	1:1000 (WB)
Phospho-ERK1/2 (Thr202/Tyr204)	Cell Signaling	9101	1:1000 (WB)
DAPI	Sigma-Aldrich	D9542	1:1000

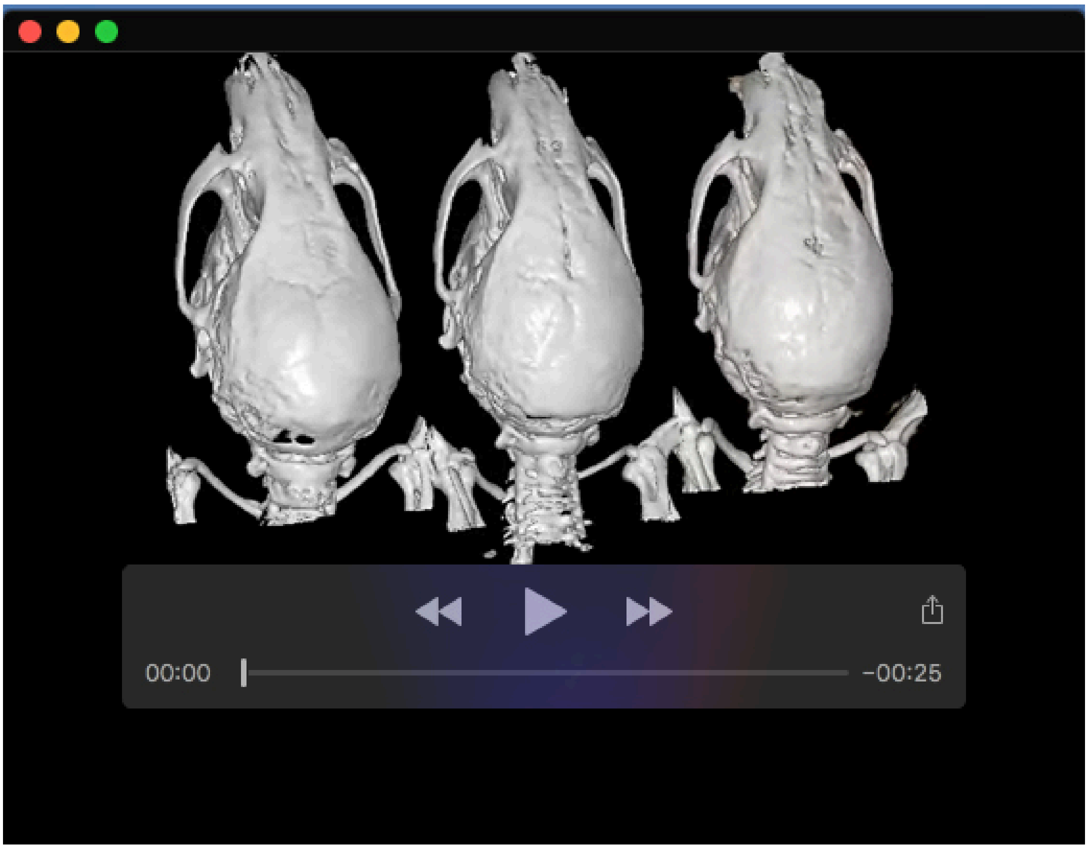

Movie 1. *hUSP8^{GOF}* expression induces cranial skeletal defects.

3D-rendered micro-CT reconstruction of control (left), *hUSP8$^{GOF/+}$;Sox2^{Cre}* (middle) and *hUSP8$^{GOF/GOF}$;Sox2^{Cre}* (right) mouse skulls.

Disease Models & Mechanisms • Supplementary information
